# Enhanced Cutaneous Delivery of Quercetin Using Poloxamer 407–Cellulose Derivative Systems: A Topical Approach Aimed at Melanoma Therapy

**DOI:** 10.1021/acsomega.6c06908

**Published:** 2026-10-13

**Authors:** Gabrielli Furlan, Jéssica Bassi da Silva, Lidiane Vizioli de Castro Hoshino, Mauro Luciano Baesso, Fernanda Fogagnoli Simas, Marcos Luciano Bruschi

**Affiliations:** † Laboratory of Research and Development of Drug Delivery Systems, Postgraduate Program in Pharmaceutical Sciences, Department of Pharmacy, State University of Maringa, 87020-900 Maringa, PR, Brazil; ‡ Department of Physics, State University of Maringa, 87020-900 Maringa, PR, Brazil; § Laboratory of Inflammatory and Neoplastic Cells, Cell Biology Department, Section of Biological Sciences, Federal University of Parana, 80060-000 Curitiba, PR, Brazil

## Abstract

Skin cancer therapy still presents limitations regarding efficacy and invasiveness, which highlights the need for alternative therapeutic approaches. Quercetin has emerged as a promising natural compound because of its antioxidant, anti-inflammatory, and potential antitumor effects. However, its clinical topical application is limited by low bioavailability. The use of thermoresponsive and bioadhesive polymeric systems constitutes a promising strategy for topical administration. This study aimed to investigate formulations composed of poloxamer 407 and cellulose derivatives containing quercetin for the treatment of skin cancers. Therefore, the systems were evaluated regarding in vitro quercetin release profile, ex vivo skin permeation, micelle morphology, formulation toxicity using *Artemia salina*, cytotoxicity, and effects on cell proliferation using fibroblasts and melanoma cells. The formulations showed prolonged release, promoting controlled quercetin release over time, and the ability to permeate deeper skin layers. The hydrodynamic diameter was within the nanometric scale, and the systems exhibited a homogeneous micellar distribution. None of the formulations were cytotoxic or cytostatic in both fibroblasts and melanoma cell lines. The F2-Q4 altered cellular metabolic activity in melanoma cells, suggesting a modulation of cellular metabolism. Interestingly, this effect was not observed in nontumoral fibroblasts. Further studies are required to elucidate the mechanisms involved and investigate the potential of this system for topical melanoma treatment.

## Graphical abstract

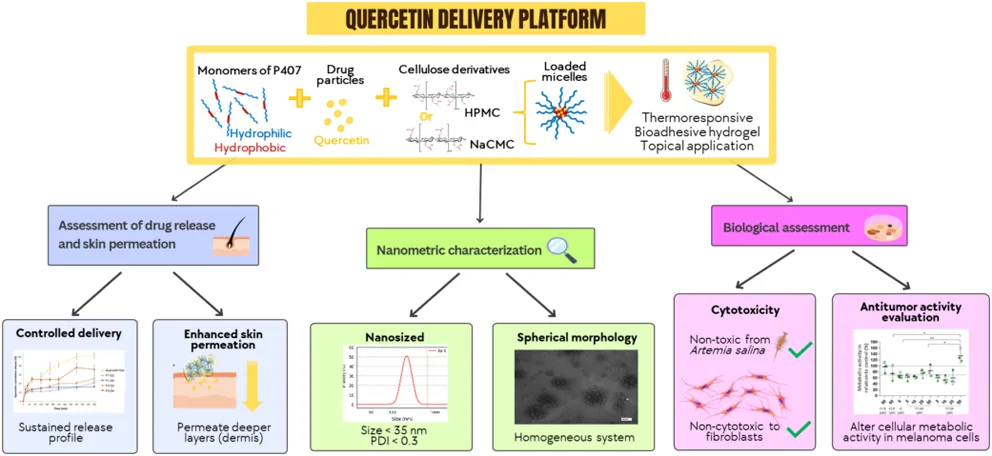

## 1 Introduction

Nowadays, topical therapies can be used as complementary approaches for the treatment of skin cancer^1^ and have emerged as promising options because they offer fewer invasive treatments with lower systemic toxicity,^2,3^ compared to standard therapies. Furthermore, as few effective agents are available and less invasive therapies are particularly desirable, especially for melanoma.^4^

Phytochemicals have been administered either alone or in combination with conventional chemotherapeutic agents, coloaded in nanostructured carriers to enhance therapeutic efficiency against cancer.^5^ These combinations have demonstrated synergistic effects in conventional treatment.^6^ In particular, flavonoids constitute a group of phenolic compounds found in a wide variety of plants. These compounds contribute to the sequestration of reactive oxygen species (ROS) and are capable of preventing disease development as promising chemoprevention agents. Their antioxidant properties help to protect cells from oxidative DNA damage induced by the oxidative stress associated with cancer.^5,7^

In addition to their antioxidant activity, increasing evidence has demonstrated the antitumor potential of flavonoids against skin cancers. For instance, a phenolic compound-rich extract exhibited cytotoxic activity against human melanoma cells (cell line A375) and induced apoptosis through both intrinsic and extrinsic signaling pathways, highlighting its therapeutic potential in melanoma treatment.^8^ Therefore, flavonoids have been extensively investigated as natural bioactives in the development of new therapies for the prevention and/or treatment of skin cancer and may therefore serve as potential therapeutic adjuvants.^7,9,10^

Quercetin is a flavonoid of the flavonol class that displays strong antioxidant activity. It is commonly administered topically due to its numerous promising effects on skin health.^10^ Owing to its anti-inflammatory properties, quercetin reduces chronic inflammation and accelerates the wound-healing process, promoting tissue regeneration.^11^ It also exhibits antitumor activity and has been shown to reduce metastasis in several tumor types.^12^

Treatment of melanoma using quercetin has been shown to inhibit cell growth, decrease proliferative activity, induce apoptosis, and reduce both tumor volume and metastasis in in vitro and in vivo models.^13,14^ Quercetin has demonstrated potential as an effective active pharmaceutical ingredient for melanoma therapy, helping to prevent the progression to more aggressive forms.^12^ The antitumor activity of this flavonoid is strongly linked to its antioxidant properties. It has been associated with inhibition of cell proliferation and the activation of apoptosis.^15^

However, the precise mechanisms underlying the therapeutic effects of quercetin against malignant melanoma have not yet been fully elucidated^15^ and remain under extensive investigation, particularly regarding its therapeutic potential and its incorporation into topical formulations designed to improve its therapeutic performance.^16^

Despite its promising pharmacological properties, the therapeutic application of quercetin is hindered by its low oral bioavailability. Therefore, topical administration has emerged as an attractive strategy to maximize its beneficial effects.^12^ In this context, polymeric platforms in the treatment of skin and mucosal cancer can help to localize drug delivery to affected cells^17^ and increase the concentration of the biologically active agent at the target site.^4,18,19^ In particular, polymeric hydrogels can be applied locally, thereby reducing adverse effects on healthy cells.^20^

There is growing interest in developing nanoformulations based on quercetin-loaded nanoparticles for the treatment of skin cancer.^21^ Nano-based carriers like titanium dioxide nanoparticles, nanoemulgels, lipid carrier gels, and PLGA TPGS nanoparticles have been proposed for primarily the enhancement of quercetin’s stability, bioavailability, and anticancer activity. These systems are valuable for mechanistic studies. However, they often rely on complex fabrication steps, organic solvents, or excipients with less established dermatological safety profiles.

The development of thermoresponsive and bioadhesive polymeric platforms has gained prominence due to their ability to respond to thermal stimuli and adhere to biological surfaces such as the skin, thereby prolonging residence time at the application site and reducing the frequency of administration.^22–27^ These systems are characterized by their physical or reversible cross-linking capacity, which enables an easy transition from solution to gel.^28^ Their responsiveness to the environment allows for improved retention at the site of application, as well as sustained and controlled drug release.^29^

Polymeric platforms composed of cellulose derivatives combined with poloxamer 407 (P407) have received attention due to this copolymer’s ability to form micelles and encapsulate hydrophobic drugs.^30,31^ In a previous study by our research group, this combination demonstrated the ability to incorporate quercetin into its micellar matrix, resulting in formulations with mechanical, physicochemical, solution–gel transition, rheological, and bioadhesive properties, constituting a distinct and complementary strategy specifically optimized for topical cutaneous delivery. First, these systems are thermoresponsive, which allows easy application, followed by in situ gelation and prolonged residence on the skin. Second, they exhibit bioadhesive properties, a feature not commonly observed in other nanobase formulations.^21^ Moreover, P407–cellulose derivative systems provide a solvent-free micellar solubilization strategy; incorporating quercetin directly into a cold P407 solution enabled homogeneous formulations without precipitates, achieving quercetin loading four times greater than its solubility in water. This contrasts with several nano-based systems that require organic solvents or more elaborate preparation methods. The use of well-established, biocompatible excipients (P407 and cellulose derivatives) also offers a translational advantage, as these polymers are widely used in approved topical products and have excellent safety profiles. Furthermore, the presence of quercetin significantly improved the evaluated parameters compared to those without the bioactive ingredient, highlighting the potential of this platform for the topical administration of quercetin.^32^

In this context, this study aimed to investigate a thermoresponsive, bioadhesive polymeric platform containing quercetin for the treatment of skin cancer, presenting it as a potential alternative for topical management of cutaneous melanoma. To support this hypothesis, a series of investigations were conducted, including in vitro release and ex vivo skin permeation studies and micelle morphology analysis. The toxicity of the polymeric platform containing quercetin was assessed using *Artemia salina* and cytotoxicity and viability assays were performed using nontumoral fibroblasts and melanoma cells.

## 2 Materials and Methods

### 2.1 Materials

Poloxamer 407 (P407) and quercetin (purity degree ≥95%, MW: 302.24 g/mol) were purchased from Sigma-Aldrich (Sao Paulo, SP, Brazil). Hydroxypropyl methylcellulose (HPMC; K100; Methocel; with 8.1% hydroxypropoxyl and 22% methoxyl content) was kindly donated by Colorcon Dow Chemical Company (Dartford, U.K.). Sodium carboxymethylcellulose (NaCMC; degree of substitution between 0.8 and 0.95) was purchased from Synth (Diadema, SP, Brazil). Purified water was obtained in-house using a water purification system (Evoqua Water Technologies, Pittsburgh, PA). All reagents were used without further purification.

### 2.2 Preparation of Formulations

The aqueous formulations were prepared using fixed concentrations of 17.5% (w/w) P407 and 0.3% (w/w) NaCMC (F1-B) or HPMC (F2-B).^31,33,34^ A methodology adapted from the cold method described by Schmolka was used.^35^ The cellulose derivative was dispersed in purified water, after which P407 was added, and the mixture was left to stand in the refrigerator for 48 h. The preparation was then subjected to magnetic stirring at 5 °C until complete homogenization.

The quercetin formulations were prepared by incorporating quercetin into P407^32^. First, P407 was dissolved in half of the required amount of purified water and allowed to swell at room temperature for 48 h. Quercetin (0.02% or 0.04%, w/w) was then added to the P407 aqueous solution, and the preparation was stirred until full homogenization. In the remaining half of the purified water, the cellulose derivative (NaCMC or HPMC) was dissolved, and the mixture was added to the previous phase (Table 1). All formulations were then transferred to amber glass flasks and stored at room temperature for at least 24 h prior to further analysis.

**Table 1:** Composition of the Polymeric Formulations Composed of Poloxamer 407 (P407), Sodium Carboxymethylcellulose (NaCMC) or Hydroxypropyl Methylcellulose (HPMC), with or in the Absence of Quercetin

	Composition (%, w/w)			
Formulations	P407	NaCMC	HPMC	Quercetin
F1-B	17.5	0.3		
F1-Q2	17.5	0.3		0.02
F1-Q4	17.5	0.3		0.04
F2-B	17.5		0.3	
F2-Q2	17.5		0.3	0.02
F2-Q4	17.5		0.3	0.04

### 2.3 In Vitro Evaluation of Drug Release Profile

Quercetin content in the formulations was determined using a previously validated UV–vis spectrophotometric method. The wavelength used was λ = 375 nm and the percentage recovery was calculated based on the standard calibration curve for quercetin in methanol (*y* = 0.0693*x* – 0.0197; *R* ^2^ = 0.9983). To determine the actual content of the formulations, they were diluted in methanol, and the quercetin content in each formulation was quantified; the percentage recovery was subsequently calculated.

The solubility of quercetin in the aqueous dissolution medium containing 2% (w/w) P407 was determined to ensure sink conditions. An excess amount of quercetin (150 μg/mL) was added to the medium, shaken vigorously on a shaker for 24 h, and then left to stand at room temperature (25 °C) for an additional 24 h. Afterward, a sample was collected, filtered through a 0.45 μm PTFE membrane (13 mm, Bionaky, China), and diluted in methanol prior to UV/vis spectrophotometric analysis to determine the maximum soluble concentration in the medium.

The in vitro release of quercetin was evaluated using a vertical diffusion cell (50 mL) apparatus based on a modified Franz’s model equipped with temperature control, maintained at 34 ± 0.5 °C. The donor chamber had a surface area of 4.5 cm^2^, and a cellulose acetate membrane (molecular weight cutoff 12 400 Da; Sigma-Aldrich, Sao Paulo, SP, Brazil) was used as the support, previously hydrated in purified water for at least 12 h. A volume of 50.0 mL of dissolution medium composed of purified water and 2% (w/w) P407 was used to ensure sink conditions, and the medium was maintained under constant magnetic stirring.

The samples were placed on the membrane. Approximately 0.625 g of the formulation containing 0.02% (w/w) quercetin and 0.3125 g of the formulation containing 0.04% (w/w) quercetin were weighed. For this purpose, 5.0 mL of a stock solution containing 0.025 mg/mL quercetin previously prepared with the dissolving medium was used.

During each assay, 1.0 mL of dissolution medium was collected at time intervals of 15 min, 30 min, 1 h, 2 h, 3 h, 4 h, 6 and 8 h and immediately replaced by fresh dissolution medium.^33^ The sample was quantified using a previously validated UV–vis spectrophotometric method at λ = 375 nm for quercetin quantification, and readings were carried out right after collection at room temperature. All analyses were conducted with at least three replicates for each sample.

The release kinetics of quercetin, as well as the transport mechanism from the systems, were evaluated using the general Korsmeyer–Peppas model, which describes the drug release from polymeric matrix systems,^36,37^ as shown in the eq 1:

1 $$\frac{M_{t}}{M_{\infty }}=k\cdot t^{n}$$

where *M_t_*/*M*_∞_ = fraction of drug released; *k* = release kinetic constant; *t* = release time; *n* = exponent that can indicate the drug release mechanism.

### 2.4 Analysis of Skin permeation by Photoacoustic Spectroscopy

Porcine ear skin was obtained from a local slaughterhouse for human consumption, and its use did not require institutional ethical approval. They were collected from young, albino, and freshly slaughtered animals. The ears were washed, the hair was trimmed, and the tissue of the dorsal region was dissected to get the dermis and epidermis. Only ears with intact skin (free of warts, spots, or lesions) were used.^38^

First, the spectra of the formulations (F1-Q2, F1-Q4, F2-Q2 and F2-Q4) were obtained in the ultraviolet and visible regions, with a wavelength range of 250–500 nm. To evaluate skin permeation, 50 mg of each was placed onto a defined 1 cm^2^ area of the epidermal surface and gently spread using a spatula to form a continuous and visually uniform layer covering the entire delimited area. After 30 min of application, the sample was analyzed using photoacoustic spectroscopy equipment consisting of a 1000W xenon arc lamp (Oriel, model 68820) with a light source at a power of 800 W.

Light was diffracted as it passed through the 3.16 mm inlet and outlet slits of the monochromator (Oriel, model 77250). A mechanical modulator (Stanford Research Systems, model SR 540) was then used to modulate the light at 13 Hz, directing it onto the sample. Higher-order diffraction was eliminated using bandpass filters. The treated porcine skin sample was placed inside the photoacoustic cell and sealed with a transparent quartz window (2 mm thick and 8 mm in diameter). Pressure variations generated by the periodic heating of the sample produced the photoacoustic signal, which was captured by a capacitive microphone (model 2669, Kjær). A lock-in amplifier (model 5110, EG&G Instruments) was also employed.

The thickness of the tissue contributing to the photoacoustic signal was determined by calculating the thermal diffusion length (μ_s_). This parameter is defined according to eq 2:

$$\mu_{s}=\sqrt{\frac{D}{\pi_{f}}}$$ 2

where μ_s_ is the thermal diffusion length, *D* is the thermal diffusivity and *f* is the light modulation frequency. Using *D* = 4.1 × 10^–4^ cm^2^/s^39^ for the thermal diffusivity of the tissue (epidermis and dermis), the resulting μ_s_ was 32 μm. This confirms that the readings were obtained near the surface on which the light was incident, considering that the average sample thickness was approximately 788 μm.

The sample was then turned over so that the opposite side could be analyzed. Detection of the characteristic band on the opposite surface indicates that the quercetin permeated through the epidermis of the porcine skin.^39,40^

### 2.5 Analysis by Dynamic Light Scattering

The hydrodynamic diameter (*D*_h_) and the polydispersity index (PDI) of the micelles of the formulations were determined using a dynamic light scattering (DLS) instrument (Litesizer 500, Anton Paar GmbH, Ostfildern, Germany). The samples were diluted in ultrapure water at a 1:10 ratio. Measurements were performed in a 1 mL cuvette, and the average micelle size was recorded at a scattering angle of 90°, at 34 °C, and analyzed using Kalliope software (Anton Paar).^33,41^

### 2.6 Transmission Electron Microscopy (TEM)

The morphological evaluation of the surface of the selected formulations was performed by using a JEM-1400 transmission electron microscope (JEOL, Tokyo, Japan) operating at an accelerating voltage of 120 kV. The selected formulations (F1-B, F1-Q4, F2-B, and F2-Q4) were diluted 100-fold in ultrapure water, placed onto copper grids, and dried at room temperature. They were then negatively stained with a 2% (w/v) uranyl acetate solution prior to imaging.^33^

### 2.7 Cytotoxicity Assessment Using *Artemia salina*

The toxicity assessment was performed using *A. salina* following the methodology previously described.^42^ The cysts were placed in a saline solution (0.1 mol/L NaCl) at room temperature and kept in a dark chamber for hatching, connected to an illuminated chamber (7 W lamp) to allow nauplii migration by phototropism. After 48 h, 1.0 mL of the suspension (containing approximately 20 nauplii) was collected and transferred to a Petri dish containing 1.0 mL of the formulation under evaluation. The number of live and dead nauplii was recorded after 3 h of exposure,^33^ and all tests were performed in three replicate samples for each formulation. The predicted viability calculation was performed according to eq 3:

3 $$viability\;\left(\%\right)=\frac{no.\;lives-no.\;dead}{no.\;total}\times 100$$

### 2.8 In Vitro Cytotoxicity and Cell Proliferation Assays

#### 2.8.1 Cell Culture and Treatment Preparation

Viability and proliferation assays were performed using the murine melanoma cell line B16-F10 (BCRJ, 0212) and Balb/3T3 clone A31 fibroblasts (ATCC, CCL-163). The cells were cultured in Dulbecco’s Modified Eagle Medium (DMEM; Gibco, Cat. 12,800-017, Boston, MA) supplemented with 10% noninactivated fetal bovine serum (FBS; Gibco, Cat. 12657029, Boston, MA), 1 U/mL penicillin and 1 μg/mL streptomycin (Thermo Fisher, Cat. 15140122, Boston, MA). Cultures were maintained at 37 °C and 5% CO_2_ until reaching 80–90% confluence for plating. For seeding, the cells were washed with PBS, detached with trypsin/EDTA (Gibco, Cat. 15400054, Boston, MA), counted using a hemocytometer, and plated in 96-well plates at densities of 500 cells/well for melanoma cells and 2000 cells/well for fibroblasts. The cells were incubated for 24 h at 37 °C and 5% CO_2_ to allow proper adhesion.

The freeze-dried formulation was exactly weighed (134 mg) and diluted in 1.0 mL of sterile DMEM culture medium, where the molar quercetin concentration was calculated to perform serial dilutions. Working solutions were prepared for each of the final desired concentrations. This procedure was performed for all of the formulations. While free quercetin was diluted in DMSO at 0.3 mg/mL for the preparation of the working solution, it was diluted in DMEM.

The treatment time was 24 h. The final quercetin concentration used for the free compound (Q) was 5 μM. For the formulations F1-Q4 and F2-Q4, they were tested at the final quercetin concentrations of 5, 10, 25, and 50 μM. For the blank formulations (F1-B and F2-B), which do not contain quercetin, the amount applied corresponded to that used in the formulations containing the highest quercetin concentration. The maximum concentration evaluated was at 50 μM, since preliminary screening with a broader concentration range revealed significant cytotoxicity of the blank formulations (F1-B and F2-B) at higher concentrations in both cell lines. This effect of blank formulations is expected, as P407-cellulose systems can alter membrane interactions and metabolic activity when applied at elevated doses in vitro. Importantly, these concentrations are substantially higher than those expected in real topical use, where dilution by the stratum corneum and limited penetration significantly reduce cellular exposure. All experiments were performed in biological triplicates with technical quadruplicates.

#### 2.8.2 Analysis of Cell Viability, Cytotoxicity, and Cell Proliferation

After 24 h of exposure to treatments described above, 100 μL/well of neutral red (NR; Sigma-Aldrich, Sao Paulo, SP, Brazil) at 0.04 mg/mL prepared in supplemented DMEM was added, and the cells were incubated at 37 °C and 5% CO_2_ for 3 h. The dye retained within the cells was then extracted using 100 μL/well of extraction solution (50% ethanol, 49% ultrapure water, and 1% glacial acetic acid). After shaking for 30 min, the absorbance was measured at λ = 540 nm using a microplate spectrophotometer (BioTek Instruments, Winooski, VT) to determine the retention rate per cell and infer cell viability.^43^

For proliferation analysis, the same cell samples previously fixed by the NR extraction solution were stained with 100 μL/well of 0.25 mg/mL crystal violet solution (CV; Electron Microscopy Sciences, Hatfield, PA) and incubated for 20 min at room temperature. The wells were then washed twice with ultrapure water, and 100 μL of 33% (v/v) acetic acid was added to elute the dye. After shaking for 30 min, the absorbance was measured at λ = 570 nm to determine cell density.^44^

A cytotoxicity assay was performed using MTT (3-[4,5-dimethylthiazol-2-yl]-2,5-diphenyltetrazolium bromide). After the treatment period, MTT solution was added to reach a final concentration of 0.05 mg/mL. The plate was incubated for an additional 3 h at 37 °C and 5% CO_2_. The medium was then removed, and 100 μL of 99.9% DMSO was added to each well to solubilize the formazan crystals. After shaking, the absorbance was measured at λ = 550 nm to quantify metabolically active cells.^45^

### 2.9 Statistical Analysis

For statistical analysis, the GraphPad Prism 5.0 program (GraphPad Software) was used, and the results were compared using Analysis of Variance (ANOVA). For ANOVA analysis, post hoc comparisons between individual group means were performed using Tukey’s Honestly Significant Difference test. A *p*-value < 0.05 was considered statistically significant.

## 3 Results and Discussion

### 3.1 Drug Content

To determine the actual concentration of quercetin present in the formulations, the samples were diluted in methanol and analyzed to quantify their quercetin content. Accurate dosing of the active pharmaceutical ingredient (API) is essential to ensure product quality and homogeneity. For very small amounts of API, acceptable concentration limits typically fall within ±10% of the actual content.^46^ The actual content values obtained for each formulation were then used as reference points for subsequent quantitative analyses. The percentage of quercetin recovered was higher in the NaCMC formulations compared with the HPMC ones, ranging from 89.47% to 98.86% (Table 2). However, only F1-Q4 demonstrated satisfactory recovery within the 98–102% range recommended by ICH Q2­(R1).^47^

**Table 2:** Determination of Quercetin Content in Formulations

Formulations	Actual content (%, w/w)	Recovery (%, w/w)
F1-Q2	0.0193 ± 0.0002	96.74 ± 1.00
F1-Q4	0.0395 ± 0.0001	98.86 ± 0.27
F2-Q2	0.0179 ± 0.0002	89.47 ± 1.20
F2-Q4	0.0381 ± 0.00004	95.21 ± 0.09

### 3.2 Solubility of Quercetin in the Dissolution Medium

The solubility obtained from the saturated solution after 24 h at room temperature (25 °C) was 30.3591 ± 3.5394 μg/mL of quercetin in aqueous 2% (w/w) P407 solution. This value was used to calculate the sink conditions for the release assays. The solubility of API in aqueous media can be improved through the use of cosolvents, such as ethanol at different concentrations,^48^ or surfactants like Tween 80 at low concentrations,^49^ to ensure adequate sink conditions during dissolution and release assays. In this study, a 2% (w/w) P407 solution was selected as the dissolution medium because it enhanced quercetin solubility.

### 3.3 In Vitro Evaluation of Drug Release Profile

The study of the drug release profile of a formulation is a key strategy in the development of therapeutic systems, as it enables the investigation of both the release kinetics and the underlying transport model.^36^ The analysis was performed under controlled temperature and stirring conditions for all formulations, as well as for free quercetin, to monitor and understand how quercetin is released into the dissolution medium from the selected polymeric platforms, according to the methodology employed.

The quercetin release profiles over time (Figures 1 and S1) show that the formulations exhibited a slow and controlled release compared with free quercetin, which dispersed 100% of the drug present in the donor medium within 8 h. In contrast, the formulations released less than 40% of the active ingredient during the first 4 h, except for formulation F2-Q4, which released approximately 50% within the first hour.

**Figure 1:**
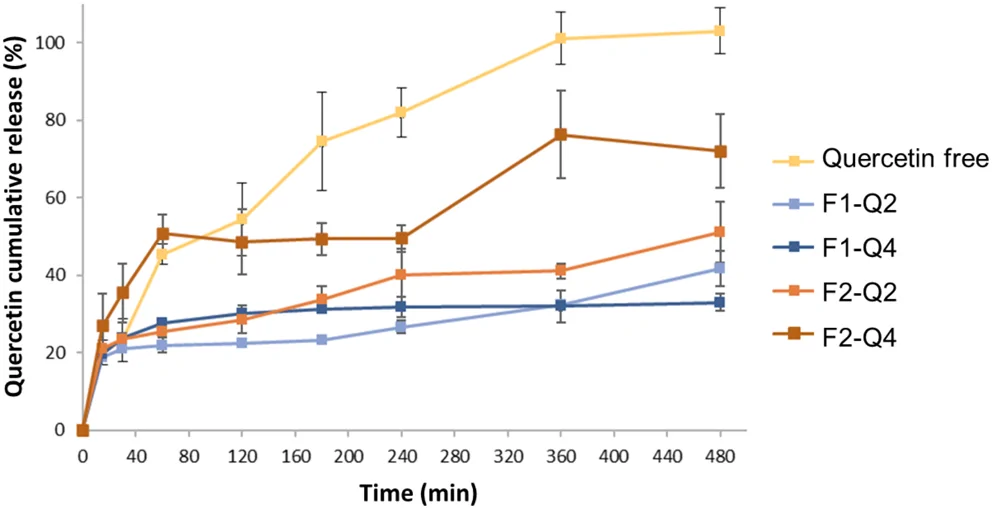
In vitro release profile of quercetin from the formulations in aqueous dissolution medium containing poloxamer 407 (2%, w/w).

Polyphenolic compounds generally exhibit strong binding affinity to polymeric matrices.^50,51^ The delayed release observed for the formulations, compared with free quercetin, can be attributed to the localization of the active ingredient within the core of the poloxamer micelles.^52^ The differences observed between the more concentrated formulations (F1-Q4 and F2-Q4) may result from distinct interactions established between quercetin and each cellulose derivative (NaCMC and HPMC), as previously reported. These findings indicate that quercetin forms stronger molecular interactions with NaCMC/P407 than HPMC/P407 systems.^32^

Additionally, a cellulose acetate membrane (molecular weight cutoff of 12,400 Da) was used as the support for the sample and influenced the quercetin release profile of the formulations. The hydrodynamic diameter (*D*_h_) of the micelles obtained in this study ranged from 21.3 to 35.3 nm (Table 4), a value far greater than the retention pore size of this membrane. Consequently, quercetin must first be released from the micelles and then diffuse through the membrane in its free form to reach the receptor compartment. This process would artificially reduce the apparent release rate of quercetin. Therefore, the measured release rate may also reflect the transmembrane diffusion rate of quercetin rather than solely its actual release from the hydrogel matrix.

The mechanism of drug release from the formulations was evaluated using the general Korsmeyer–Peppas equation (Table 3), which describes the kinetic model of drug release and the transport mechanism governing this process.^53,54^

**Table 3:** Release Rate Coefficients for Quercetin In Vitro Release Data Fitted with Korsmeyer–Peppas Release Model^a^

	Parameters		
Formulations	*k* (%.min^–1^)	*n*	*r*
F1-Q2	14.56 ± 1.00	0.10 ± 0.02	0.8857 ± 0.1004
F1-Q4	13.10 ± 2.18	0.13 ± 0.04	0.9496 ± 0.0760
F2-Q2	14.48 ± 0.36	0.14 ± 0.01	0.8960 ± 0.0159
F2-Q4	18.06 ± 1.45	0.22 ± 0.10	0.7256 ± 0.0502

a Value evaluated for up to 60% of quercetin released; *r* = correlation coefficient.

The Korsmeyer–Peppas model showed correlation coefficient (*r*) results, ranging from 0.7256 to 0.9496. The value of the release exponent (*n*) determines the mechanism involved in drug release and allows its classification. For planar geometry (semisolid systems), when *n* ≤ 0.5, transport occurs by Fickian diffusion; when *n* = 1, transport is non-Fickian (Case-II transport). Anomalous transport occurs when 0.5 < *n* < 1 and is governed by both diffusion and polymer chain swelling or relaxation.^36,53,54^ In this study, all formulations presented n-values from 0.1 to 0.22, indicating a modified release profile governed by Fickian diffusion. In this mechanism, drug release depends solely on the diffusion of the permeant into the formulation, the dissolution of quercetin, and its subsequent diffusion through the polymeric matrix.^42^ F2-Q4 formulation fitted weakly for the Korsmeyer–Peppas model (r = 0.7256) and the n-values near 0.1 may limit the interpretation using this model. Nonetheless, the transmembrane diffusion rate can also influence this behavior.

Diffusion within polymeric hydrogels regulates the rate of drug release by acting as a barrier that slows drug exit, promotes sustained release over extended periods, and balances protection against loss or degradation with efficient delivery at the target site.^55^

Analysis of the release rate constant (*k*) showed values ranging from 13.10 to 18.06 min^–1^, indicating differences in drug release kinetics among systems. Formulations with lower k values presented slower release, likely due to the greater retention of quercetin within the polymer matrix compared to free quercetin. These findings are consistent with those of the in vitro release profiles. Among all of the formulations, F2-Q4 showed the fastest release, which can be attributed to its composition containing HPMC and a higher quercetin concentration. This suggests that HPMC-based formulations facilitate quercetin release more readily at higher loadings. In contrast, NaCMC-based formulations demonstrated a slower release profile, particularly at higher concentrations of quercetin.

These differences arise from the distinct functional groups present in each cellulose derivative, which interact differently with P407.^31,33^ It is known that HPMC, in the presence of quercetin, interacts more strongly with the hydrophilic segments of P407,^32^ potentially facilitating the dissolution of quercetin from the micellar core into the aqueous medium. A slow and prolonged release profile is desirable, as it may reduce the frequency of administration or even enable a single application on the skin, ultimately improving patient adherence to treatment.^56^

### 3.4 Ex Vivo Skin Permeation Analysis by Photoacoustic Spectroscopy

The skin permeation was evaluated by photoacoustic spectroscopy using porcine ear skin, which exhibits high similarity to human skin, is easily accessible, and is more suitable for permeation tests. Porcine ear skin is widely recognized as one of the closest nonhuman analogues to human skin in terms of anatomical structure, lipid composition, and barrier function. Its characteristics (stratum corneum thickness, follicular density, and permeability) closely resemble those of human skin.^57,58^ It is a well-established and ethically accessible model for dermal and transdermal research. In contrast, mouse skin, although commonly used, presents significantly higher permeability and a thinner stratum corneum, which may overestimate permeation rates and reduce the translational relevance of the results.^59^

Permeation studies by photoacoustic spectroscopy allow the assessment of drug retention within the skin following topical administration as well as the ability of the compound to cross the stratum corneum barrier and remain in this tissue to exert its biological activity. The porcine skin was analyzed after 30 min of application to assess the ability of the formulations to promote early penetration through the skin and their detection in the dermal region rather than to characterize the kinetics of skin permeation. The results are displayed in Figures 2 and S2. Formulations exhibited two broad optical absorption bands centered at approximately 257 and 379 nm (Figure 2A). The detection of these characteristic bands throughout the tissue thickness served as an indicator of the presence of topically applied substances. The optical absorption spectra of the epidermal surface 30 min after the application (Figure 2B) showed these bands for all formulations when compared with the control epidermis, with greater intensity observed for the more concentrated formulations (F1-Q4 and F2-Q4).

**Figure 2:**
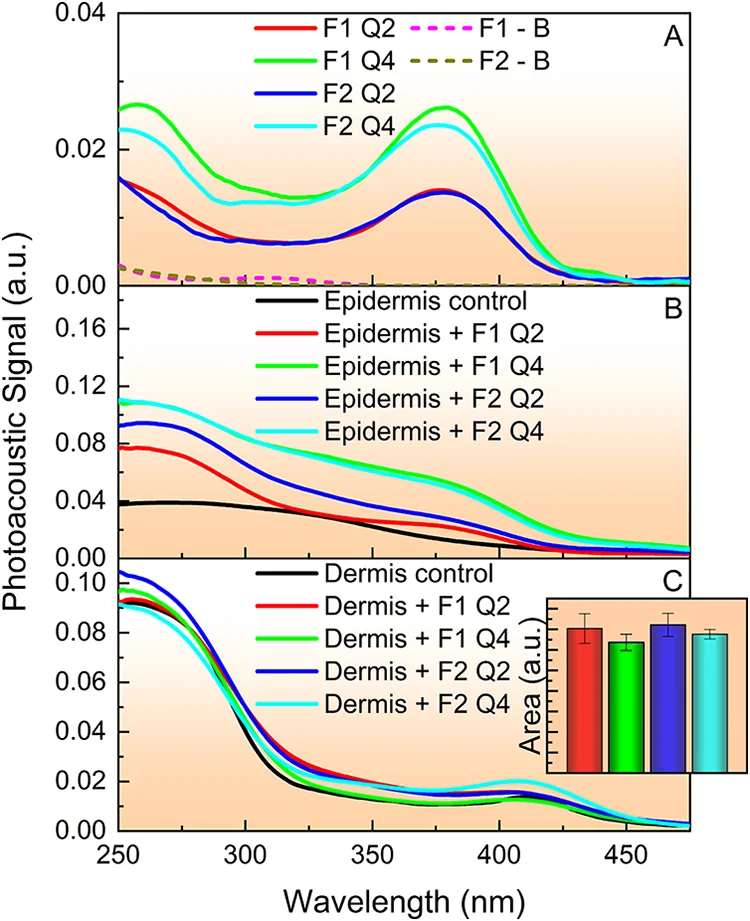
Optical absorption spectra of the formulations obtained by photoacoustic spectroscopy: (A) Optical absorption spectra of the formulations; (B) epidermis; (C) dermis with the characteristic absorption area of drugs in the dermis. F1-B and F2-B are the formulations without quercetin. The inset represents the area under the curve obtained by means of an integral in the spectral region of λ = 250–500 nm.

The optical absorption spectra of the dermal surface 30 min after application of the formulations (Figure 2C) showed increased band intensity in the characteristic regions, indicating the presence of the formulations within the dermis. The band presented at 415 nm in the control dermis is characteristic of the presence of blood vessels in the tissue, and the samples with formulation show a slight increase in this region.^60^ To determine the skin permeation, the area under the absorption curve was integrated into the 250–500 nm spectral range. All formulations could reach the dermis, and although no significant differences in the permeation rate were observed among them, a slight increase was noted for the more concentrated formulations.

Although formulation F2-Q4 demonstrated a higher quercetin release capacity over time, this characteristic did not influence the permeation results, as the ex vivo test was performed after 30 min of contact within the formulations. Skin permeation is a dynamic process, and evaluating it at a single time point does not allow determination of the permeation kinetics or assessment of changes in cutaneous drug distribution over time. To address this limitation, future studies should incorporate multiple permeation time points to better characterize the temporal profiles of skin permeation.

### 3.5 Analysis by Dynamic Light Scattering (DLS)

The hydrodynamic diameter (*D*_h_) and polydispersity index (PDI) of the formulations, with or without quercetin, were determined by DLS at 34 °C (Table 4).

**Table 4:** Hydrodynamic Diameter (*D*_h_) and Polydispersity Index (PDI) of Micelles from the Polymeric Systems, with and without Quercetin, Evaluated at 34 °C

Formulations	*D*_h_ (nm)	PDI
F1-B	23.4 ± 0.5	0.22 ± 0.01
F1-Q2	22.6 ± 0.6	0.15 ± 0.04
F1-Q4	30.1 ± 0.9	0.23 ± 0.00
F2-B	21.3 ± 0.2	0.10 ± 0.01
F2-Q2	35.3 ± 2.5	0.22 ± 0.02
F2-Q4	28.1 ± 1.1	0.23 ± 0.01

All analyses were performed at this temperature to mimic the physiological temperature of the application site (skin) and to ensure micelle formation. The gelling temperature previously reported for these formulations was about ∼30 °C.^32^ Under these conditions, the P407 chains form micellar structures suitable for measurement.^61^

The formulations exhibited variations in micellar hydrodynamic diameter (*D*_h_), ranging from 21.3 to 35.3 nm, with significant differences between the NaCMC- and HPMC-based systems. The smallest sizes were observed in the formulations without quercetin (*p* < 0.05). This increase in the micellar diameter in the quercetin-loaded systems suggests incorporation of the flavonoid into the micellar core.

The differences observed between the cellulose derivatives used in this study arise from the distinct ways in which HPMC and NaCMC interact with P407, leading to more- or less-structured systems. As previously reported,^33^ micelles formed by P407 in the presence of different cellulose derivatives vary in size. HPMC-based systems tend to form smaller micelles, whereas NaCMC-based systems form micelles with a larger core and in a lower amount. These findings are consistent with the results obtained here for formulations without quercetin.

Despite these differences, the *D*_h_ results are within the expected range for poloxamer nanomicelles,^30,62^ confirming that P407 is capable of forming nanoscale micellar structures. Nanomicelles are known to enhance drug penetration into the skin, and smaller particle sizes generally facilitate deeper permeation into cutaneous layers.^63^

The PDI varied according to quercetin concentration in both systems, with the greatest variation observed in the formulations containing 0.04% quercetin (*p* < 0.05). The formulations with the smallest micellar diameter also exhibited the lowest PDI values. Nonetheless, all formulations presented PDI of <0.3, indicating that the micelles were uniformly dispersed within the system. Such values are characteristic of stable and homogeneous samples.^64^

### 3.6 Transmission Electron Microscopy (TEM)

Subsequently, the formulations without quercetin (F1-B and F2-B) and those containing the highest concentration of quercetin (F1-Q4 and F2-Q4) were analyzed by TEM to assess their morphology (Figures 3 and S3). The TEM images revealed spherical micelles arranged in clusters, a feature attributed to the dilution of the formulations during preparation.

**Figure 3:**
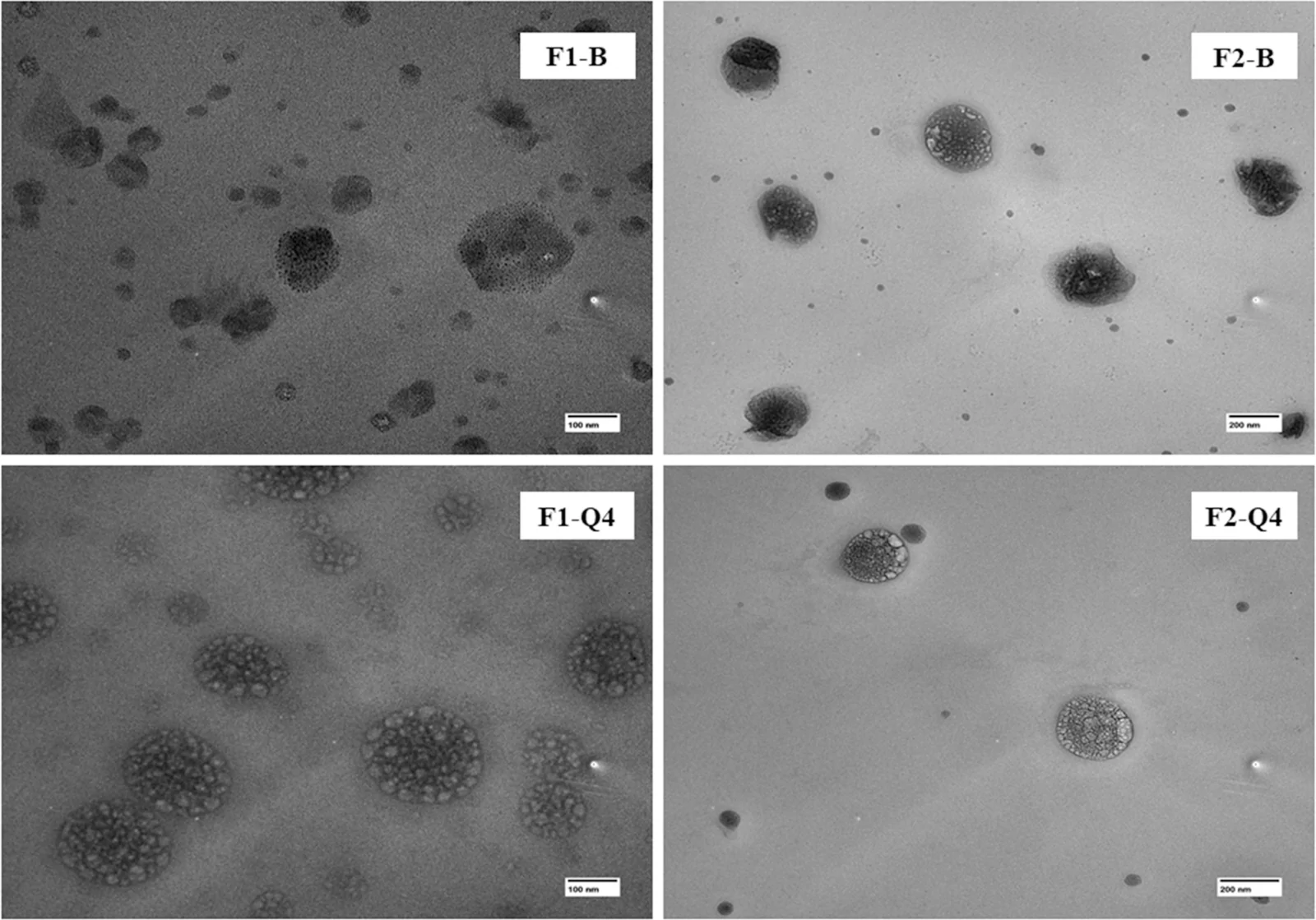
Images obtained by Transmission Electron Microscopy (TEM) of the selected formulations F1-Q4 and F2-Q4, along with their corresponding quercetin-free systems (F1-B and F2-B). A scale bar of 100 nm was used for the F1 formulations (F1-B and F1-Q4), and a scale bar of 200 nm was used for the F2 formulations (F2-B and F2-Q4).

### 3.7 Evaluation of Toxicity Using *Artemia salina*

*A. salina* is a small crustacean commonly used to assess the general toxicity of bioactive agents and is considered a preliminary screening model. This marine microcrustacean exhibits high sensitivity to a wide range of compounds, making it an accessible and efficient assay, comparable to tests performed with human cell lines.^65^ This toxicity assay was employed to estimate the lethality of the formulations, with or without quercetin, against *A. salina*.

The formulations were not lethal, even after 3 h of exposure, with the viability remaining close to 100% for all samples. Therefore, neither quercetin nor the polymeric system exhibited toxicity under these conditions. Other authors have also employed this assay to evaluate the toxicity of semisolid pharmaceutical systems containing different active agents, as it is an economical, rapid, and reliable method. In addition, the amount of formulation used in the test is essential for superior performance, since large amounts can cause immobilization of the nauplii due to the high consistency of the formulation. Viability values above 85% are generally considered acceptable, whereas lower values may indicate potential toxicity.^30,33,42^

### 3.8 Tumor and Nontumor Cell Viability and Proliferation following Treatment with the Formulations

Formulations F1-Q4 and F2-Q4 at four concentration levels of quercetin (5, 10, 25, and 50 μM) and quercetin (Q) at 5 μM were evaluated. Free drug was investigated at 5 μM due to its poor aqueous solubility even using DMSO. Higher quercetin concentrations (including 50 μM) resulted in marked precipitation in the culture medium and could result in significant nonspecific cytotoxicity unrelated to its biological activity. However, the F1-Q4 and F2-Q4 formulations successfully solubilized quercetin at an equivalent nominal concentration of 50 μM due to the micellar encapsulation provided by P407, allowing to investigate higher quercetin levels without precipitation.

Formulations with and without quercetin did not alter melanoma tumor cells (B16-F10) and nontumor fibroblasts (Balb/3T3) viability under the evaluated conditions (Figures 4 and S4). The neutral red assay showed that the treatments did not cause substantial disruption of lysosomal integrity (Figure 4C,D), indicating the absence of irreversible cell death.^43^ This result is consistent with the quercetin release profile from the formulations, which demonstrated a sustained and gradual drug release rather than an immediate burst that could produce acute cytotoxic effects.

**Figure 4:**
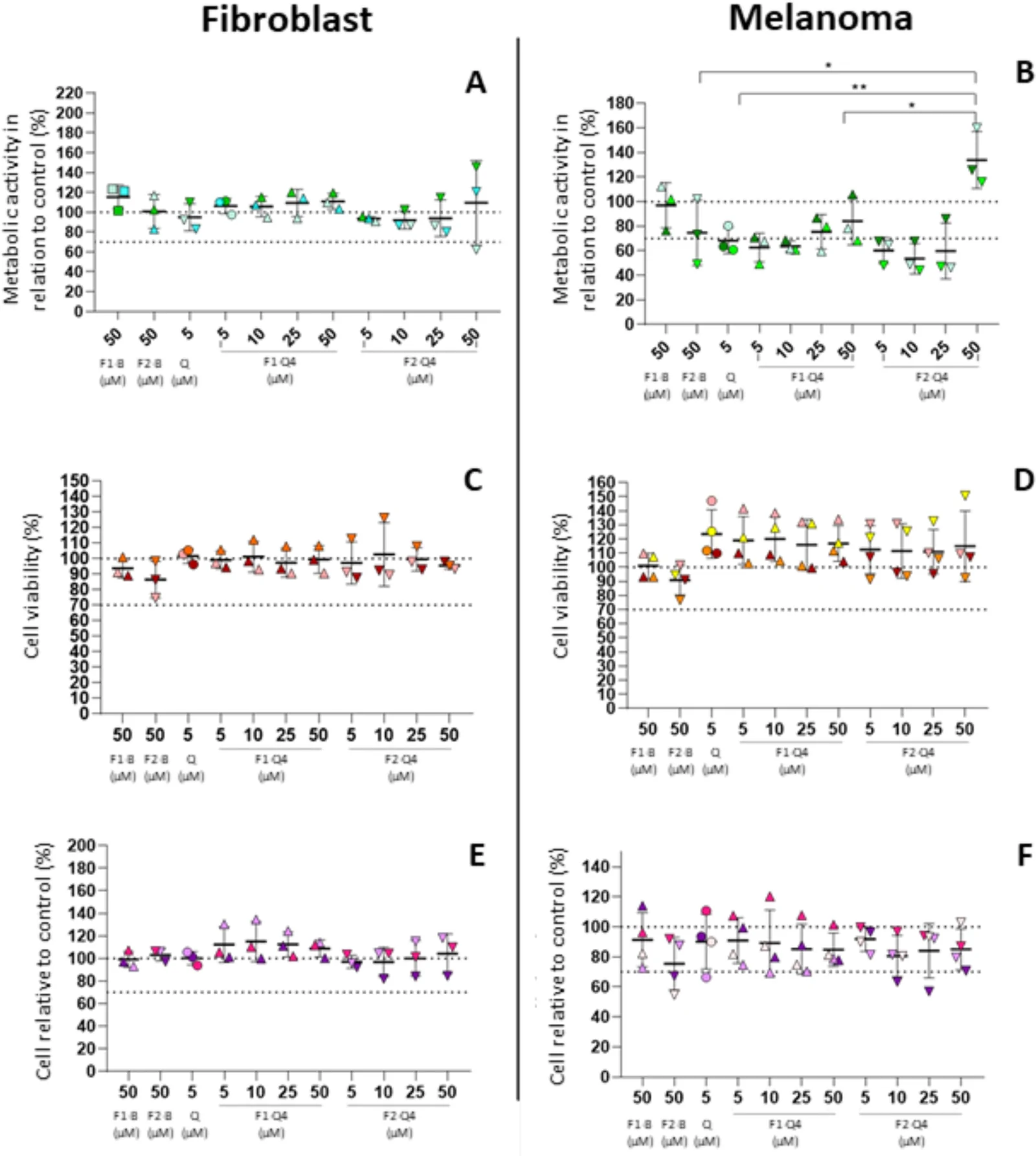
Analysis of viability and cell proliferation of fibroblast and melanoma cells in the presence of free quercetin and quercetin-loaded formulations: (A, B) MTT metabolic activity assay; (C, D) cell viability by neutral red assay; (E, F) number of cells by violet crystal assay. The assays were carried out after 24 h of treatment. *p < 0.05, **p < 0.01 (ANOVA, Tukey–multiple comparisons between all groups).

A 35% increase in metabolic activity was observed in the melanoma cell line following treatment with F2-Q4/50 μM (Figure 4B); however, the number of cells remained unchanged (Figure 4F), indicating that no proliferation occurred.^44^ The statistically significant increase in MTT-reducing activity observed in tumor cells treated with F2-Q4/50 μM was not observed in tumor cells treated with free quercetin (Q), blank formulation (F2-B), and F1-Q4/50 μM (Figure 4A,B). This finding may reflect an effect of quercetin incorporated into the formulation on cellular metabolic processes associated with redox reactions, although direct interference with the MTT assay cannot be excluded.^45^

Quercetin is widely recognized for its antioxidant properties, which may modulate mitochondrial oxidative metabolism.^66^ The F2-Q4/50 μM formulation induced an increase in MTT-reducing activity, whereas the number of viable cells remained unchanged. The faster release of quercetin from F2-Q4 during the first hours of treatment may have contributed to this response; however, further studies are required to determine the mechanisms underlying this effect.

The literature indicates that quercetin can induce cellular senescence or act as a senolytic agent, depending on the cell type and concentration, a feature that may be relevant in the context of antitumor therapies.^67–69^ Senescence has been investigated as a potential strategy against cancer progression due to its ability to induce cell-cycle arrest.^70^ However, its role in cancer biology is complex, as it can exert tumor-suppressive effects but may also contribute to tumor persistence and resistance to standard therapies.^71^

Although mitochondrial alterations are not commonly used as a tool for detecting senescent cells, senescence has been associated with increased mitochondrial mass and alterations in mitochondrial morphology and function, which may result from reduced mitochondrial turnover, including impaired mitophagy.^72,73^ As senescent cells are metabolically active, they can exhibit mitochondrial dysfunctions that can alter oxidative metabolism of these cells.^74^

These alterations may contribute to changes in cellular metabolic activity and could potentially influence MTT reduction without necessarily reflecting an increase in cell proliferation. In the present study, the increased MTT-reducing activity observed following F2-Q4/50 μM treatment, in the absence of a corresponding increase in viable cell number, may be compatible with such metabolic alterations.

However, since specific markers of senescence and mitochondrial function were not evaluated, these findings should not be interpreted as evidence of a senescent phenotype. Further studies are required to determine whether senescence or other metabolic mechanisms contribute to the effects observed following F2-Q4/50 μM treatment. It is essential to show complementary hallmarks rather than relying on a single experiment. Recommended assays include SA-β-galactosidase activity, expression analysis of p16, p21, and p53, assessment of the proliferation marker Ki-67, cell-cycle profiling by flow cytometry, EdU or BrdU incorporation assays, and morphological evaluation of enlarged/flattened senescent cells.

Future studies should also investigate the antitumor potential of this delivery system more comprehensively. They include testing higher quercetin equivalent concentrations, extending exposure times, and generating dose–response curves comparing free quercetin and formulated quercetin. Additional assays such as apoptosis and necrosis quantification, mitochondrial membrane potential analysis, ROS production, clonogenic survival, and 3D melanoma spheroid models will be very useful to evaluate more thoroughly the cytotoxic and antiproliferative effects.

## 4 Conclusions

All formulations were nanostructured, with micellar hydrodynamic diameters on the nanometric scale, and presented a homogeneous micellar population. The in vitro release profile of quercetin from the formulations was slow and governed by Fickian diffusion, consistent with the characteristics of the developed systems. Formulations composed of P407 and NaCMC exhibited slower release kinetics, whereas those containing P407 and HPMC showed faster release. All formulations permeated the epidermis and were able to reach deeper layers, with quercetin being detected in the dermis. None of the formulations showed cytotoxic or cytostatic effects on the tested cells. However, F2-Q4 (50 μM) increased MTT-reducing activity in tumor cells without a corresponding increase in cell number, indicating a modulation of cellular metabolic activity. Further studies are required to determine the mechanisms underlying this metabolic response. Additionally, these studies will help determine the potential of this polymeric formulation for topical melanoma treatment.

## Supplementary Material



## Notes

Full uncropped images of Figures 1–4. (PDF)

## References

[1] Swetter S. M., Tsao H., Bichakjian C. K., Curiel-Lewandrowski C., Elder D. E., Gershenwald J. E., Guild V., Grant-Kels J. M., Halpern A. C., Johnson T. M., Sober A. J., Thompson J. A., Wisco O. J., Wyatt S., Hu S., Lamina T. (2019). Guidelines
of Care for the Management of Primary Cutaneous Melanoma. J. Am. Acad. Dermatol..

[2] Senapati S., Mahanta A. K., Kumar S., Maiti P. (2018). Controlled Drug Delivery
Vehicles for Cancer Treatment and Their Performance. Signal Transduction Targeted Ther..

[3] Hafeez A., Kazmi I. (2017). Dacarbazine Nanoparticle Topical
Delivery System for the Treatment
of Melanoma. Sci. Rep..

[4] Cullen J. K., Simmons J. L., Parsons P. G., Boyle G. M. (2020). Topical Treatments
for Skin Cancer. Adv. Drug Delivery Rev..

[5] Mohapatra P., Singh P., Singh D., Sahoo S., Sahoo S. K. (2022). Phytochemical
Based Nanomedicine: A Panacea for Cancer Treatment, Present Status
and Future Prospective. OpenNano.

[6] Ashrafizadeh M., Zarrabi A., Hashemi F., Moghadam E. R., Hashemi F., Entezari M., Hushmandi K., Mohammadinejad R., Najafi M. (2020). Curcumin in Cancer Therapy: A Novel
Adjunct for Combination
Chemotherapy with Paclitaxel and Alleviation of Its Adverse Effects. Life Sci..

[7] Ng C. Y., Yen H., Hsiao H. Y., Su S. C. (2018). Phytochemicals
in Skin Cancer Prevention
and Treatment: An Updated Review. Int. J. Mol.
Sci..

[8] Rungjiraphirat R., Cheepchirasuk N., Suriyaprom S., Tragoolpua Y. (2025). Inhibitory
Efficacy of Arthrospira Platensis Extracts on Skin Pathogenic Bacteria
and Skin Cancer Cells. Biology.

[9] Aghamohammadi M., Zolghadr L., Nezhad N. S., Ahmadpour Yazdi H., Esfahani A. J., Gheibi N. (2022). Investigating the Effects
of Quercetin
Fatty Acid Esters on Apoptosis, Mechanical Properties, and Expression
of ERK in Melanoma Cell Line (A375). Life Sci..

[10] Wadhwa K., Kadian V., Puri V., Bhardwaj B. Y., Sharma A., Pahwa R., Rao R., Gupta M., Singh I. (2022). New Insights
into Quercetin Nanoformulations for Topical Delivery. Phytomed. Plus.

[11] Esposito L., Barbosa A. I., Moniz T., Lima S. C., Costa P., Celia C., Reis S. (2020). Design and
Characterization of Sodium
Alginate and Poly­(Vinyl) Alcohol Hydrogels for Enhanced Skin Delivery
of Quercetin. Pharmaceutics.

[12] Caro C., Pourmadadi M., Eshaghi M. M., Rahmani E., Shojaei S., Paiva-Santos A. C., Rahdar A., Behzadmehr R., García-Martín M. L., Díez-Pascual A. M. (2022). Nanomaterials
Loaded with Quercetin as an Advanced Tool for Cancer Treatment. J. Drug Delivery Sci. Technol..

[13] Ferrer P., Asensi M., Segarra R., Ortega A., Benlloch M., Obrador E., Varea M. T., Asensio G., Jordá L., Estrela J. M. (2005). Association between
Pterostilbene and Quercetin Inhibits
Metastatic Activity of B16 Melanoma. Neoplasia.

[14] Kim S. H., Yoo E. S., Woo J. S., Han S. H., Lee J. H., Jung S. H., Kim H. J., Jung J. Y. (2019). Antitumor and Apoptotic
Effects of Quercetin on Human Melanoma Cells Involving JNK/P38 MAPK
Signaling Activation. Eur. J. Pharmacol..

[15] Salgado M. T. S. F., Fernandes de Silva E., do Nascimento M. A., Lopes A. C., de Paiva L. S., Votto A. P. S. (2023). Potential
Therapeutic
Targets of Quercetin in the Cutaneous Melanoma Model and Its Cellular
Regulation Pathways: A Systematic Review. Nutr.
Cancer.

[16] Hoinoiu T., Dumitrascu V., Pit D., Schipor D. A., Jabri-Tabrizi M., Hoinoiu B., Petreus D. E., Seiman C. (2025). Quercetin as a Potential
Therapeutic Agent for Malignant Melanoma—A Review of Current
Evidence and Future Directions. Medicina.

[17] Wen, R. ; Umeano, A. C. ; Chen, P. ; Ammad, A. ; Farooqi, A. Polymer-Based Drug Delivery Systems for Cancer 2018 www.begellhouse.com. 10.1615/CritRevTherDrugCarrierSyst.2018021124 30317968

[18] Gomes F. P., Boldo E. M. (2020). Estudo de Ensaios de Permeação
e Liberação,
in Vitro, de Formas Farmacêuticas Semissólidas Tópicas
Para Padronização Dos Requisitos de Qualidade Em Uma
Indústria Farmacêutica. Res.,
Soc. Dev..

[19] Krawczyńska K., Rusak A., Górnicki T., Mrozowska M., Dzięgiel P., Gubernator J. (2026). Development
of New Liposomal Formulations
of Quercetin – in Vitro Study. Sci. Rep..

[20] Marzi M., Rostami Chijan M., Zarenezhad E. (2022). Hydrogels as Promising Therapeutic
Strategy for the Treatment of Skin Cancer. J.
Mol. Struct..

[21] Khanyabzadeh M., Emamifar A., Emtiazi N., Nazari A., Shekarriz M., Karami A., Mahallati F. H. B., Lafmejani R. N., Dariush A., Rohani S. M. M., Vadeghani E. M., Khoshnood N., Ojaghi H. R., Rezaei-Tazangi F., Arefnezhad R. (2026). Quercetin and Its Nano-Based Formulations Against Skin
Cancer: A Narrative Review. Health Sci. Rep..

[22] Abou-Shamat M. A., Calvo-Castro J., Stair J. L., Cook M. T. (2019). Modifying the Properties
of Thermogelling Poloxamer 407 Solutions through Covalent Modification
and the Use of Polymer Additives. Macromol.
Chem. Phys..

[23] Wei M., Gao Y., Li X., Serpe M. J. (2017). Stimuli-Responsive Polymers and Their
Applications. Polym. Chem..

[24] Bashir R., Majeed A., Ali T., Farooq S., Khan N. A. (2019). Floating
Oral In-Situ Gel: A Review. J. Drug Delivery
Ther..

[25] De
Souza Ferreira S. B., Moço T. D., Borghi-Pangoni F. B., Junqueira M. V., Bruschi M. L. (2016). Rheological, Mucoadhesive and Textural
Properties of Thermoresponsive Polymer Blends for Biomedical Applications. J. Mech. Behav. Biomed. Mater..

[26] Gholizadeh H., Messerotti E., Pozzoli M., Cheng S., Traini D., Young P., Kourmatzis A., Caramella C., Ong H. X. (2019). Application of a
Thermosensitive In Situ Gel of Chitosan-Based
Nasal Spray Loaded with Tranexamic Acid for Localised Treatment of
Nasal Wounds. AAPS PharmSciTech.

[27] Salah I., Shamat M. A., Cook M. T. (2019). Soluplus
Solutions as Thermothickening
Materials for Topical Drug Delivery. J. Appl.
Polym. Sci..

[28] Karoyo A. H., Wilson L. D. (2021). A Review on the Design and Hydration Properties of
Natural Polymer-Based Hydrogels. Materials.

[29] Nia S. B., Pooresmaeil M., Namazi H. (2020). Carboxymethylcellulose/Layered Double
Hydroxides Bio-Nanocomposite Hydrogel: A Controlled Amoxicillin Nanocarrier
for Colonic Bacterial Infections Treatment. Int. J. Biol. Macromol..

[30] Botan M. V. G., da Silva J. B., Bruschi M. L. (2024). Development of Nanostructured Environmentally
Responsive System Containing Hydroxypropyl Methylcellulose for Nose-to-Brain
Administration of Meloxicam. Int. J. Biol. Macromol..

[31] da
Silva J. B., dos Santos R. S., Vecchi C. F., da Silva
Souza Campanholi K., da Silva Junior R. C., de Castro Hoshino L. V., Caetano W., Baesso M. L., Simas F. F., Cook M. T., Bruschi M. L. (2023). Boosting the Photodynamic Activity of Erythrosine B
by Using Thermoresponsive and Adhesive Systems Containing Cellulose
Derivatives for Topical Delivery. Int. J. Biol.
Macromol..

[32] Furlan G., da Silva J. B., Bruschi M. L. (2025). Improvement of the Mechanical, Rheological
and Bioadhesive Properties of Environmentally Responsive Systems Containing
Different Cellulose Derivatives for Quercetin Topical Application. Mater. Today Commun..

[33] da
Silva J. B., dos Santos R. S., da Silva M. B., Braga G., Cook M. T., Bruschi M. L. (2021). Interaction between Mucoadhesive
Cellulose Derivatives and Pluronic F127: Investigation on the Micelle
Structure and Mucoadhesive Performance. Mater.
Sci. Eng. C.

[34] da
Silva J. B., Cook M. T., Bruschi M. L. (2020). Thermoresponsive
Systems Composed of Poloxamer 407 and HPMC or NaCMC: Mechanical, Rheological
and Sol-Gel Transition Analysis. Carbohydr.
Polym..

[35] Schmolka I. R. (1972). Artificial
Skin I. Preparation and Properties of Pluronic F-127 Gels for Treatment
of Burns. J. Biomed. Mater. Res..

[36] Bruschi, M. L. Strategies to Modify the Drug Release from Pharmaceutical Systems; Elsevier, 2015.

[37] Korsmeyer R. W., Peppas N. A. (1981). Effect of the Morphology of Hydrophilic Polymeric Matrices
on the Diffusion and Release of Water Soluble Drugs. J. Membr. Sci..

[38] Fernandes A. S., de Souza Ferreira S. B., de Oliveira M. C., Caetano W., Bruschi M. L. (2022). The Influence
of Different Bioadhesive Polymers on Physicochemical Properties of
Thermoresponsive Emulgels Containing Amazonian Andiroba Oil. J. Mol. Liq..

[39] Baesso M. L., Shen J., Snook R. D. (1994). Laser-Induced Photoacoustic
Signal
Phase Study of Stratum Corneum and Epidermis. Analyst.

[40] Ames F. Q., Sato F., de Castro L. V., de Arruda L. L. M., da Rocha B. A., Cuman R. K. N., Baesso M. L., Bersani-Amado C. A. (2017). Evidence
of Anti-Inflammatory Effect and Percutaneous Penetration of a Topically
Applied Fish Oil Preparation: A Photoacoustic Spectroscopy Study. J. Biomed. Opt..

[41] Gupta A., Costa A. P., Xu X., Lee S. L., Cruz C. N., Bao Q., Burgess D. J. (2020). Formulation and
Characterization of Curcumin Loaded
Polymeric Micelles Produced via Continuous Processing. Int. J. Pharm..

[42] Junqueira M. V., Borghi-Pangoni F. B., Ferreira S. B. S., Rabello B. R., Hioka N., Bruschi M. L. (2016). Functional Polymeric Systems as Delivery
Vehicles for
Methylene Blue in Photodynamic Therapy. Langmuir.

[43] Repetto G., del Peso A., Zurita J. L. (2008). Neutral
Red Uptake Assay for the
Estimation of Cell Viability/ Cytotoxicity. Nat. Protoc..

[44] Vega-Avila E., Pugsley M. K. (2011). An Overview of Colorimetric Assay Methods Used to Assess
Survival or Proliferation of Mammalian Cells. Proc. West. Pharmacol. Soc..

[45] Fotakis G., Timbrell J. A. (2006). In Vitro Cytotoxicity Assays: Comparison of LDH, Neutral
Red, MTT and Protein Assay in Hepatoma Cell Lines Following Exposure
to Cadmium Chloride. Toxicol. Lett..

[46] Uriel M., Marro D., Gómez Rincón C. (2023). An Adequate Pharmaceutical
Quality System for Personalized Preparation. Pharmaceutics.

[47] Validation of Analytical Procedures: Text and Methodology Q2(R1) International Council for Harmonisation (ICH): Geneva; 2005.

[48] Moghadam M., Seyed Dorraji M. S., Dodangeh F., Ashjari H. R., Mousavi S. N., Rasoulifard M. H. (2022). Design
of a New Light Curable Starch-Based Hydrogel
Drug Delivery System to Improve the Release Rate of Quercetin as a
Poorly Water-Soluble Drug. Eur. J. Pharm. Sci..

[49] Lu B., Huang Y., Chen Z., Ye J., Xu H., Chen W., Long X. (2019). Niosomal Nanocarriers
for Enhanced
Skin Delivery of Quercetin with Functions of Anti-Tyrosinase and Antioxidant. Molecules.

[50] de
Bezerra E. O. T., Berton S. B. R., de Oliveira A. C., Souza P. R., Vecchi C. F., Bruschi M. L., Vilsinski B. H., Martins A. F. (2021). The Cooling of Blends in Water Supports Durable, Thermo-Responsive,
and Porous Gelatin-Polyphenolic Tannin Assemblies with Antimicrobial
Activities. Mater. Today Commun..

[51] Szulc-Musioł B., Siemiradzka W., Dolińska B. (2023). Formulation
and Evaluation of Hydrogels
Based on Sodium Alginate and Cellulose Derivatives with Quercetin
for Topical Application. Appl. Sci..

[52] Dumortier G., Grossiord J. L., Agnely F., Chaumeil J. C. (2006). A Review of Poloxamer
407 Pharmaceutical and Pharmacological Characteristics. Pharm. Res..

[53] Lopes C. M., Lobo J. M. S., Costa P. (2005). Modified Release
of Drug Delivery
Systems: Hydrophilic Polymers. Rev. Bras. Ciênc.
Farm..

[54] Ritger P. L., Peppas N. A. (1987). A Simple Equation
for Description of Solute Release
II. Fickian and Anomalous Release from Swellable Devices. J. Controlled Release.

[55] Lavrentev F. V., Shilovskikh V. V., Alabusheva V. S., Yurova V. Y., Nikitina A. A., Ulasevich S. A., Skorb E. V. (2023). Diffusion-Limited Processes in Hydrogels
with Chosen Applications from Drug Delivery to Electronic Components. Molecules.

[56] De
Araújo Pereira R. R., Ribeiro Godoy J. S., Stivalet Svidzinski T. I., Bruschi M. L. (2013). Preparation and
Characterization of Mucoadhesive Thermoresponsive Systems Containing
Propolis for the Treatment of Vulvovaginal Candidiasis. J. Pharm. Sci..

[57] Summerfield A., Meurens F., Ricklin M. E. (2015). The Immunology of the Porcine Skin
and Its Value as a Model for Human Skin. Mol.
Immunol..

[58] Kong R., Bhargava R. (2011). Characterization of Porcine Skin
as a Model for Human
Skin Studies Using Infrared Spectroscopic Imaging. Analyst.

[59] Bartek M. J., LaBudde J. A., Maibach H. I. (1972). Skin Permeability
In Vivo: Comparison
in Rat, Rabbit, Pig and Man. J. Invest. Dermatol..

[60] de
Ferreira S. B. S., Slowik K. M., de Castro Hoshino L. V., Baesso M. L., Murdoch C., Colley H. E., Bruschi M. L. (2020). Mucoadhesive
Emulgel Systems Containing Curcumin for Oral Squamous Cell Carcinoma
Treatment: From Pre-Formulation to Cytotoxicity in Tissue-Engineering
Oral Mucosa. Eur. J. Pharm. Sci..

[61] Fakhari A., Corcoran M., Schwarz A., Fakhari A. (2017). Thermogelling Properties
of Purified Poloxamer 407. Heliyon.

[62] Raval A., Parmar A., Raval A., Bahadur P. (2012). Preparation
and Optimization
of Media Using Pluronic Micelles for Solubilization of Sirolimus and
Release from the Drug Eluting Stents. Colloids
Surf., B.

[63] Le
Guyader G., Do B., Rietveld I. B., Coric P., Bouaziz S., Guigner J. M., Secretan P. H., Andrieux K., Paul M. (2022). Mixed Polymeric Micelles for Rapamycin Skin Delivery. Pharmaceutics.

[64] Li B., Shemkai J. C., Zhang Q., Qiao F., Yu N., Zhao J., Zuo W., Yang J. (2025). Fabrication of an Alginate-Poloxamer
407 Thermosensitive Carboxymethyl Chitosan Stabilized Nanosuspension
Hydrogel for Enhanced Ocular Delivery of Clobetasol Propionate in
Uveitis Management. Int. J. Biol. Macromol..

[65] Rajabi S., Ramazani A., Hamidi M., Naji T. (2015). Artemia Salina as a
Model Organism in Toxicity Assessment of Nanoparticles. DARU J. Pharm. Sci..

[66] Salehi B., Machin L., Monzote L., Sharifi-Rad J., Ezzat S. M., Salem M. A., Merghany R. M., El Mahdy N. M., Klllç C. S., Sytar O., Sharifi-Rad M., Sharopov F., Martins N., Martorell M., Cho W. C. (2020). Therapeutic Potential of Quercetin: New Insights and
Perspectives for Human Health. ACS Omega.

[67] Thangasamy T., Sittadjody S., Lanza-Jacoby S., Wachsberger P. R., Limesand K. H., Burd R. (2007). Quercetin
Selectively Inhibits Bioreduction
and Enhances Apoptosis in Melanoma Cells That Overexpress Tyrosinase. Nutr. Cancer.

[68] Al
Mamun A., Sufian M. A., Uddin M. S., Sumsuzzman D. M., Jeandet P., Islam M. S., Zhang H. J., Kong A. N., Sarwar M. S. (2022). Exploring the Role of Senescence Inducers and Senotherapeutics
as Targets for Anticancer Natural Products. Eur. J. Pharmacol..

[69] Hundsberger H., Stierschneider A., Sarne V., Ripper D., Schimon J., Weitzenböck H.
P., Schild D., Jacobi N., Eger A., Atzler J., Klein C. T., Wiesner C. (2021). Concentration-Dependent
Pro- and Antitumor Activities of Quercetin in Human Melanoma Spheroids:
Comparative Analysis of 2D and 3D Cell Culture Models. Molecules.

[70] Muñoz-Espín D., Serrano M. (2014). Cellular Senescence:
From Physiology to Pathology. Nat. Rev. Mol.
Cell Biol..

[71] Singh L., Gaur H., Kaur S., Gandhi H., Samar V., Kapoor D. N. (2026). Revolutionizing Cancer Treatment with Senotherapeutics:
A Current Perspective. Cancer Chemother. Pharmacol..

[72] Vasileiou P. V. S., Evangelou K., Vlasis K., Fildisis G., Panayiotidis M. I., Chronopoulos E., Passias P. G., Kouloukoussa M., Gorgoulis V. G., Havaki S. (2019). Mitochondrial Homeostasis and Cellular
Senescence. Cells.

[73] Correia-Melo C., Marques F. D., Anderson R., Hewitt G., Hewitt R., Cole J., Carroll B. M., Miwa S., Birch J., Merz A., Rushton M. D., Charles M., Jurk D., Tait S. W., Czapiewski R., Greaves L., Nelson G., Bohlooly-Y M., Rodriguez-Cuenca S., Vidal-Puig A., Mann D., Saretzki G., Quarato G., Green D. R., Adams P. D., von Zglinicki T., Korolchuk V. I., Passos J. F. (2016). Mitochondria Are Required for Pro-ageing
Features of
the Senescent Phenotype. EMBO J..

[74] Miwa S., Kashyap S., Chini E., von Zglinicki T. (2022). Mitochondrial
Dysfunction in Cell Senescence and Aging. J.
Clin. Invest..

