# Supplementary material for: Enhanced Cutaneous Delivery of Quercetin Using Poloxamer 407–Cellulose Derivative Systems: A Topical Approach Aimed at Melanoma Therapy
Source: ACS Omega. 2026 Oct 13;11(42):65068–78. doi: 10.1021/acsomega.6c06908 (PMC13717665; doi:10.1021/acsomega.6c06908)
Supplement: Supplementary file 1 [file ao6c06908_si_001.pdf]

Enhanced Cutaneous Delivery of Quercetin using Poloxamer 407-Cellulose Derivative Systems: A Topical Approach Aimed at Melanoma Therapy

Gabrielli Furlan[a], Jéssica Bassi da Silva[b], Lidiane Vizioli de Castro Hoshino[b], Mauro Luciano Baesso[b], Fernanda Fogagnoli Simas[c], Marcos Luciano Bruschi[a,*]

[a]*Laboratory of Research and Development of Drug Delivery Systems, Postgraduate Program in Pharmaceutical Sciences, Department of Pharmacy, State University of Maringa, 87020-900, Maringa, PR, Brazil*

[b]*Department of Physics, State University of Maringa, 87020-900, Maringa, PR, Brazil*

[c]*Laboratory of Inflammatory and Neoplastic Cells, Cell Biology Department, Section of Biological Sciences, Federal University of Parana, 80060-000, Curitiba, PR, Brazil*

Supporting information

Full Uncropped Images of Figures

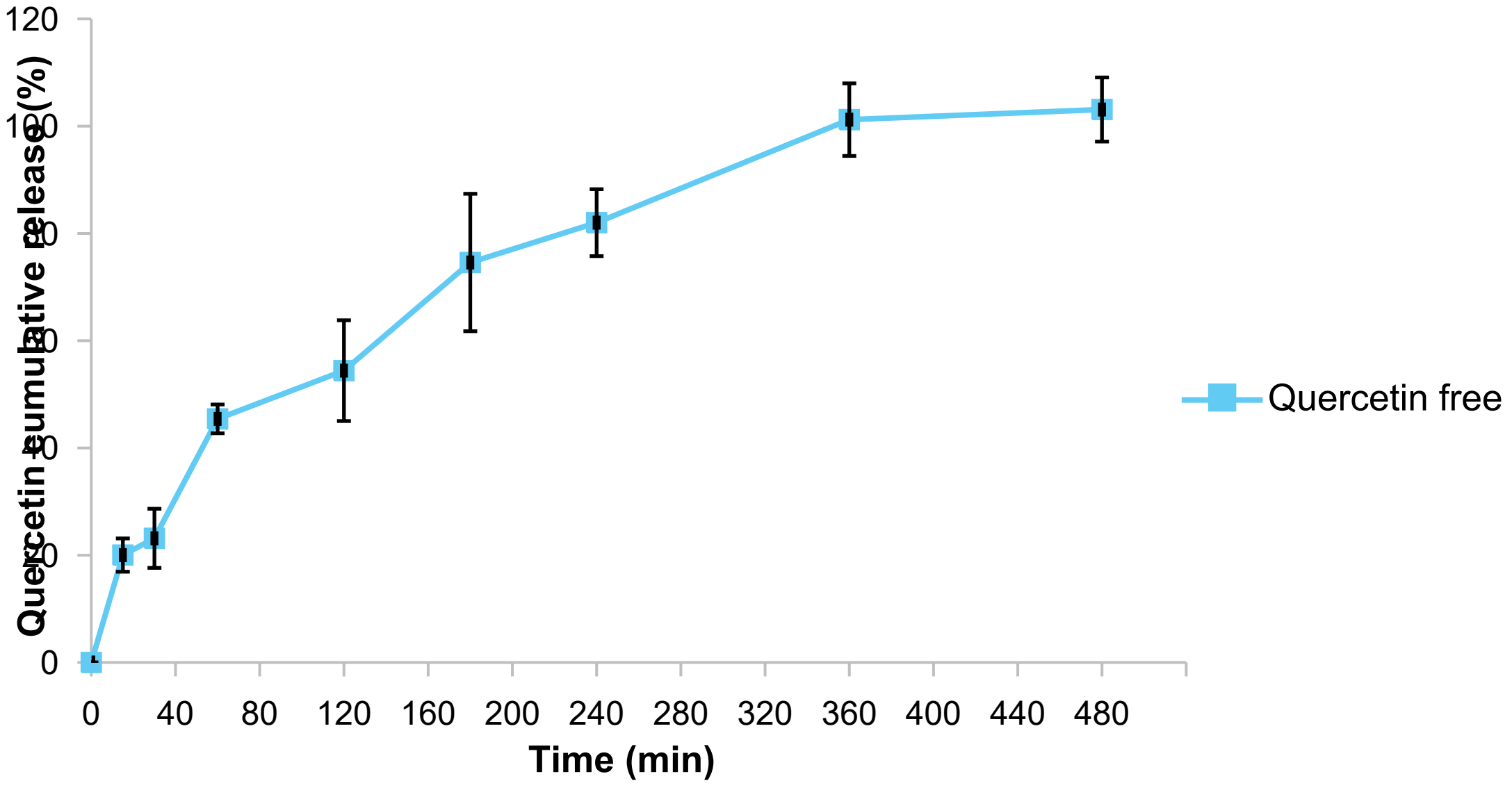
Quercetin cumulative release (%)
120
100
0
0 40 80 120 160 200 240 280 320 360 400 440 480
Time (min)
Quercetin free

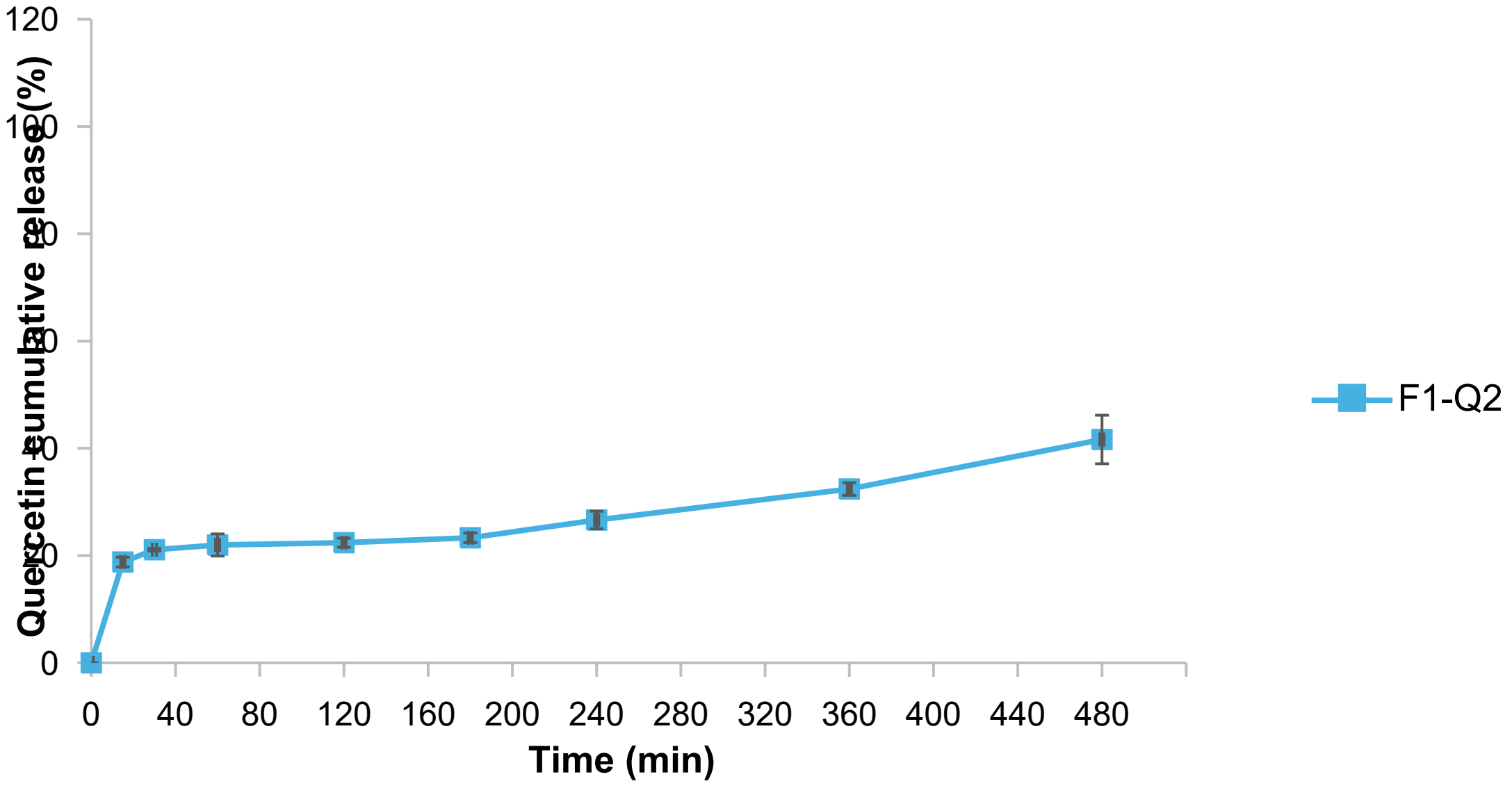
Quercetin cumulative release (%)
120
100
0
0 40 80 120 160 200 240 280 320 360 400 440 480
Time (min)
F1-Q2

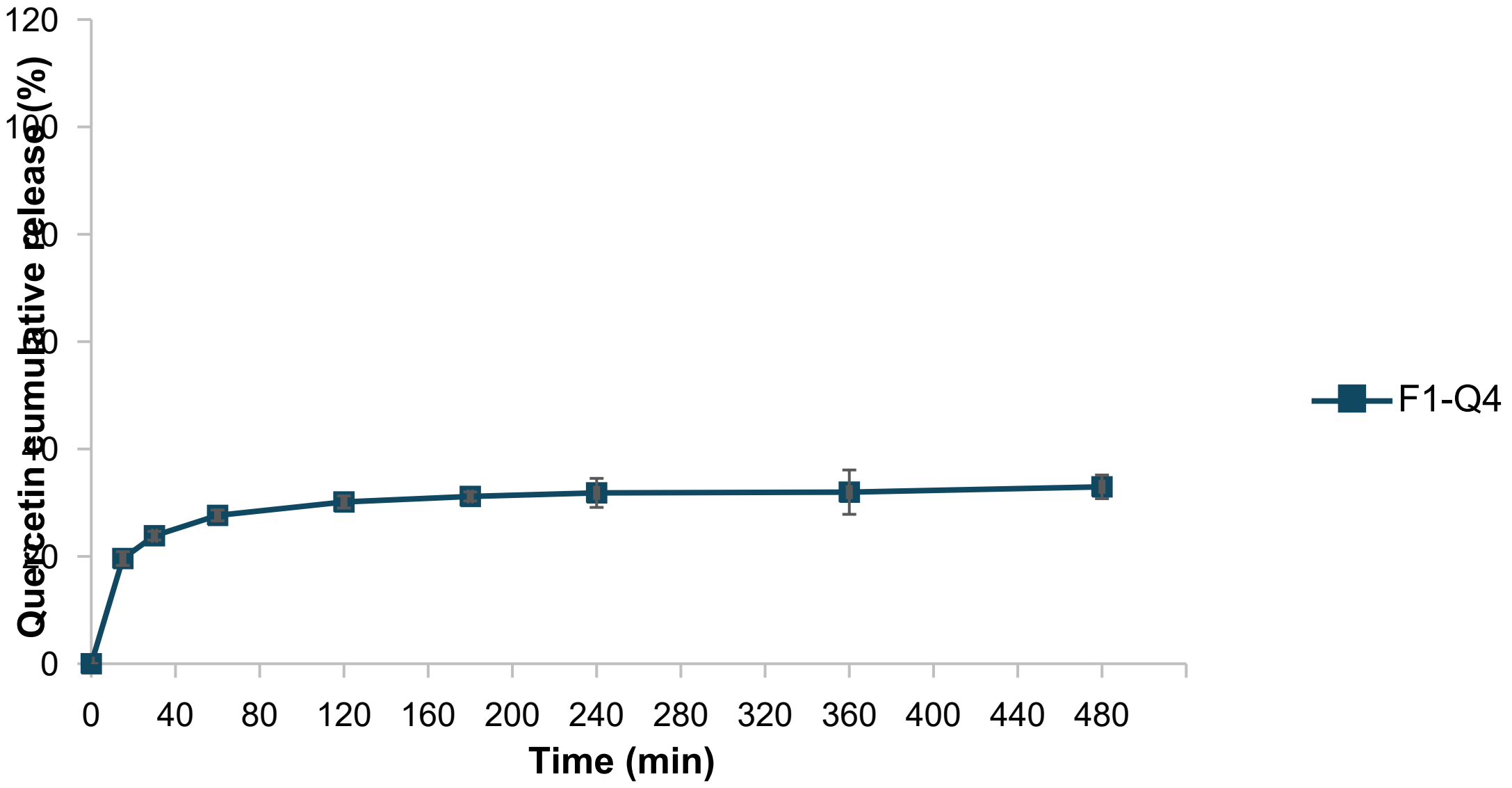
Quercetin cumulative release (%)
120
100
0
0 40 80 120 160 200 240 280 320 360 400 440 480
Time (min)
F1-Q4

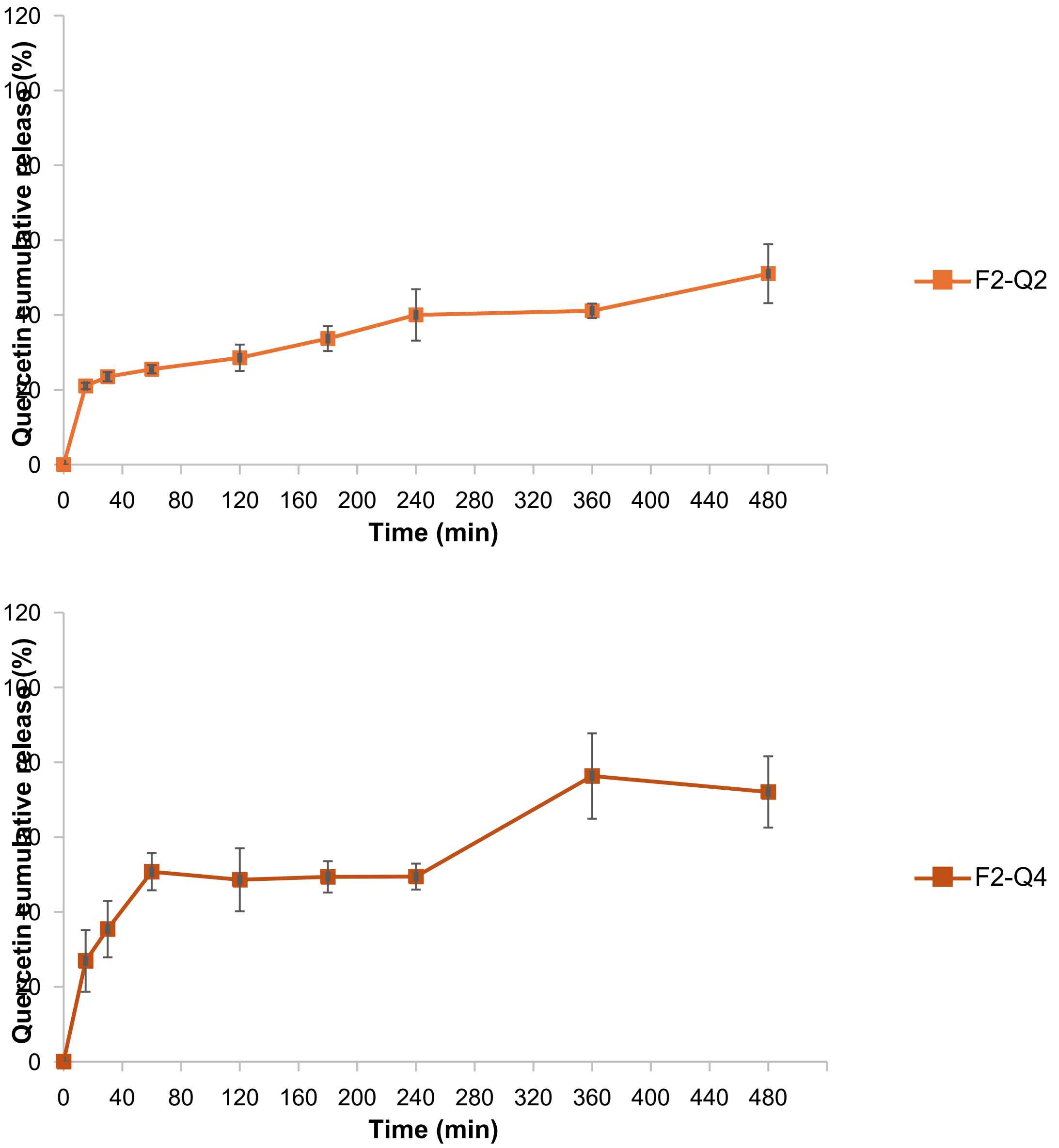

Figure S1. Separate images for each in vitro release profile of quercetin from the formulations in aqueous dissolution medium containing 2% (w/w) poloxamer 407.

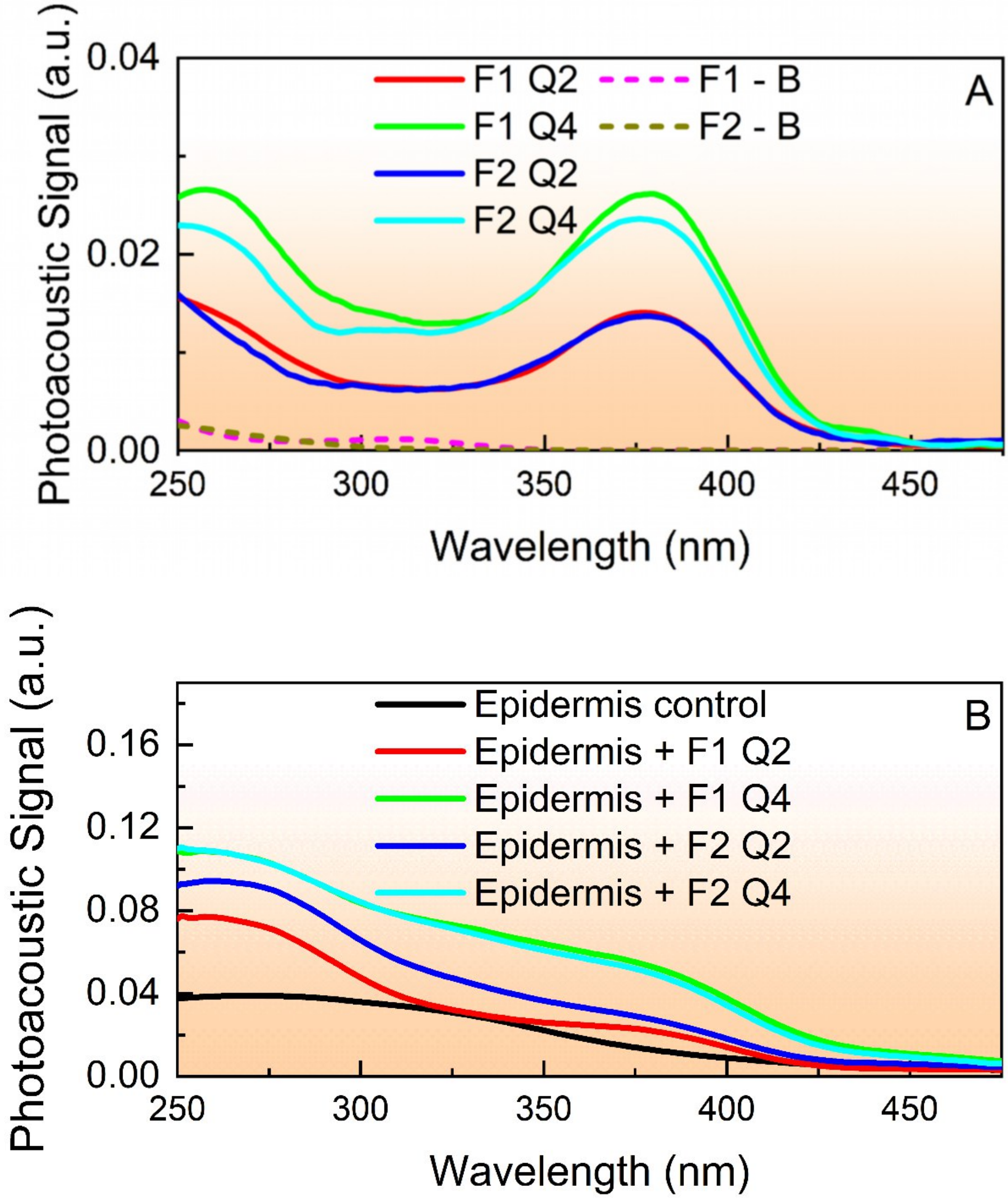

A
Photoacoustic Signal (a.u.)
0.04
0.02
0.00
250
300
350
400
450
Wavelength (nm)
F1 Q2
F1 Q4
F2 Q2
F2 Q4
F1 - B
F2 - B
B
Photoacoustic Signal (a.u.)
0.16
0.12
0.08
0.04
0.00
250
300
350
400
450
Wavelength (nm)
Epidermis control
Epidermis + F1 Q2
Epidermis + F1 Q4
Epidermis + F2 Q2
Epidermis + F2 Q4

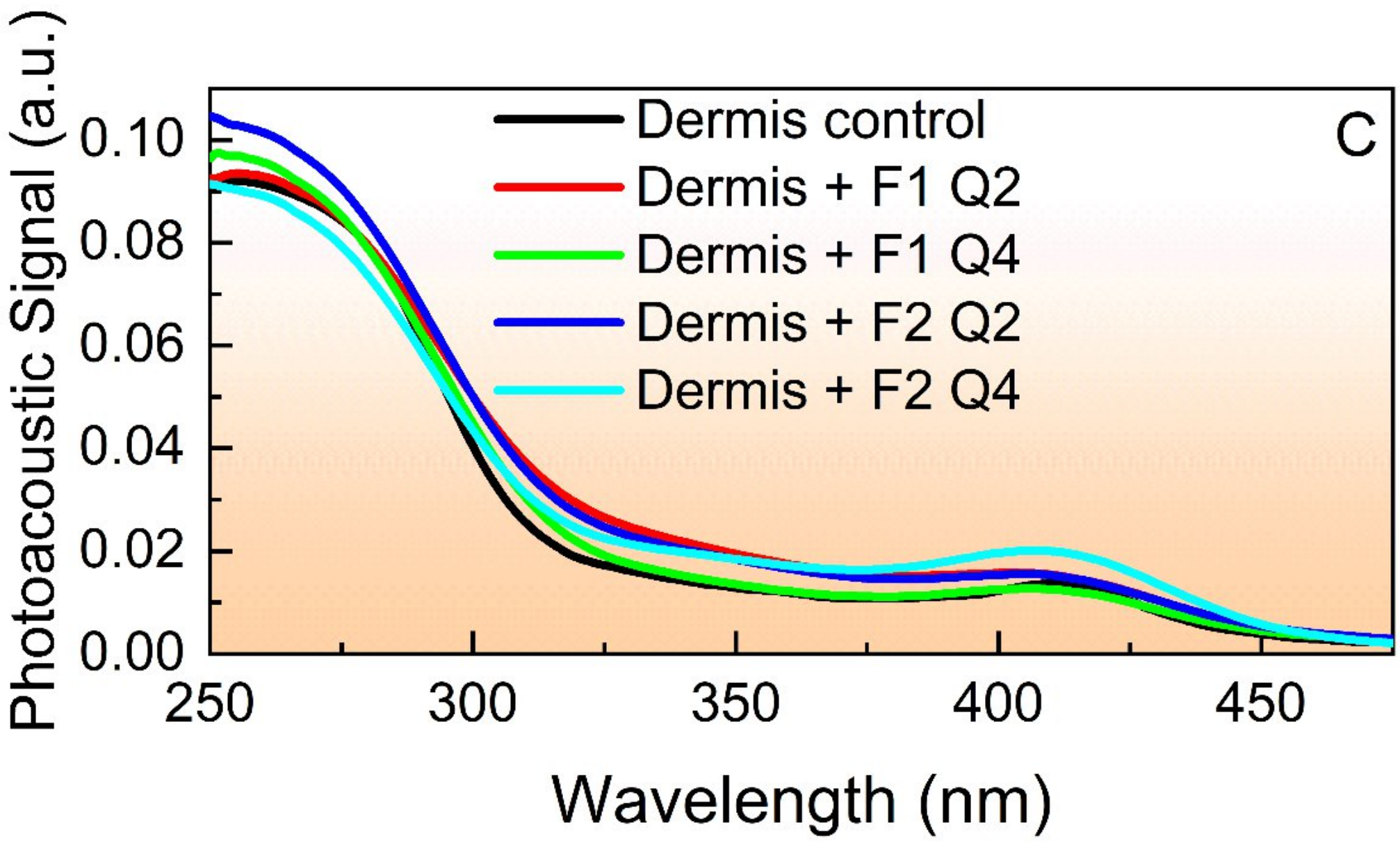

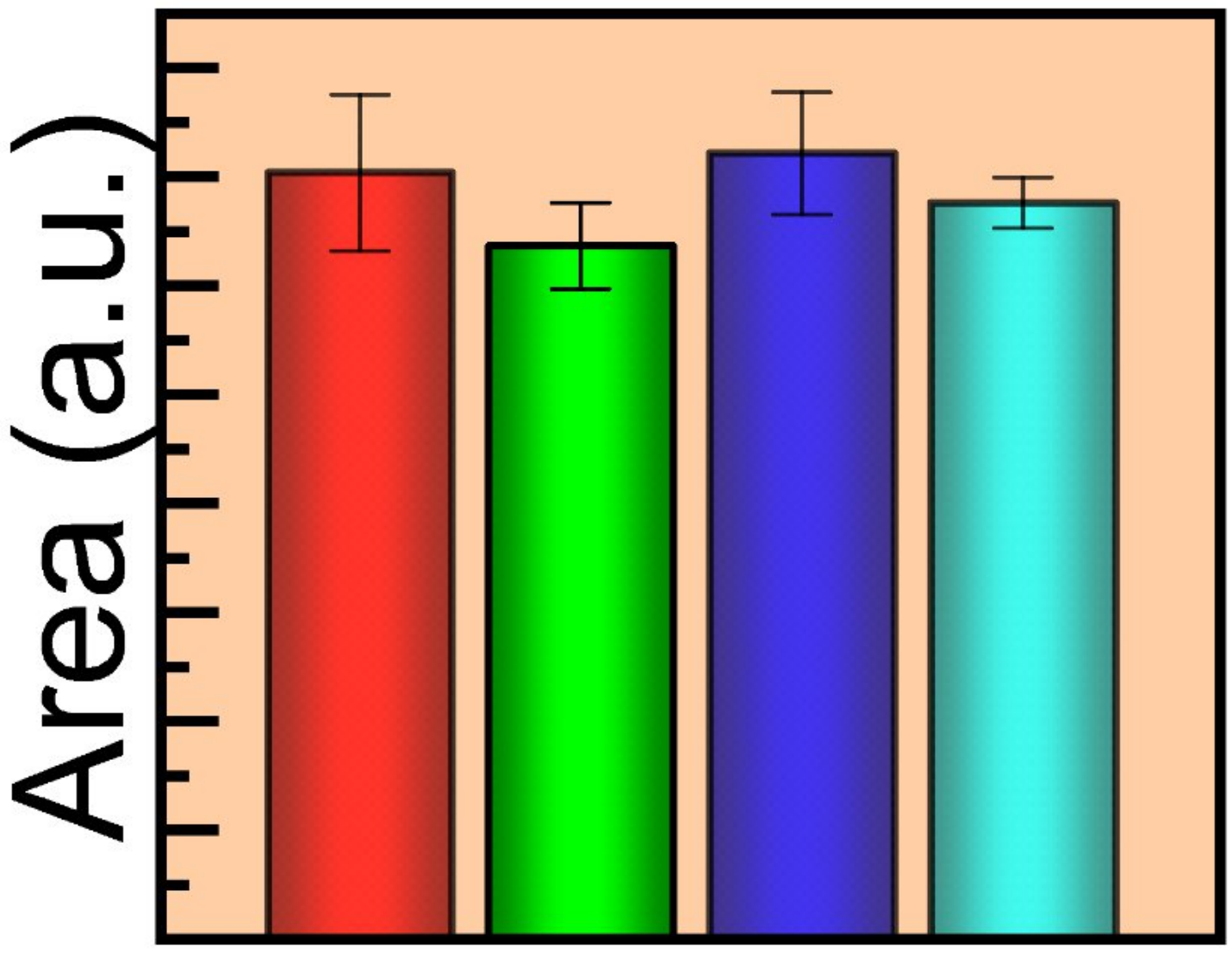

Figure S2. Separate images for optical absorption spectra of the formulations obtained by the photoacoustic spectroscopy: (A) Optical absorption spectra of the formulations; (B) Epidermis; (C) Dermis with the characteristic absorption area of drugs in the dermis. F1-B and F2-B are the formulations without quercetin. The inset represents the area under the curve obtained by means of an integral in the spectral region of λ = 250-500 nm.

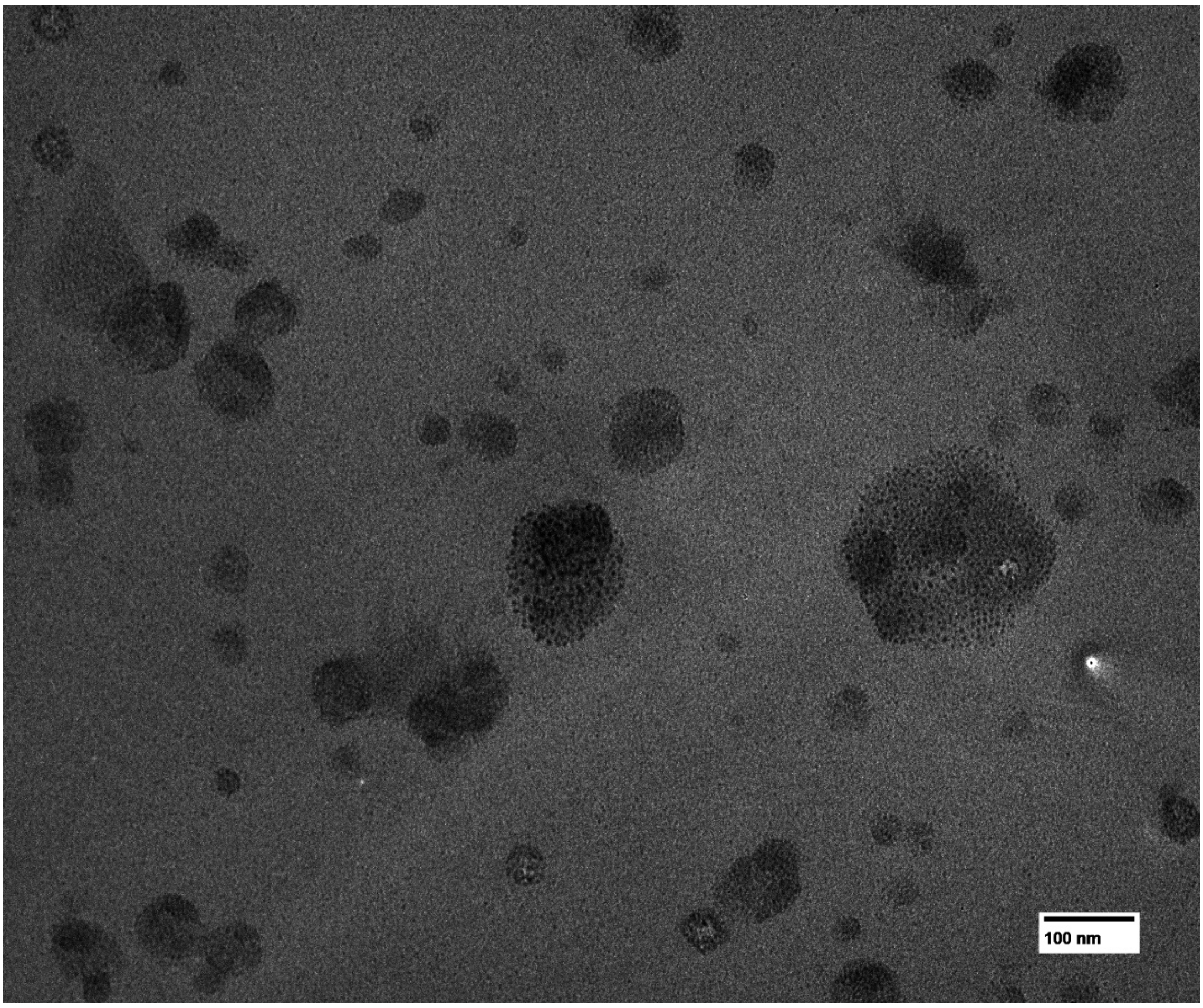

F1-B

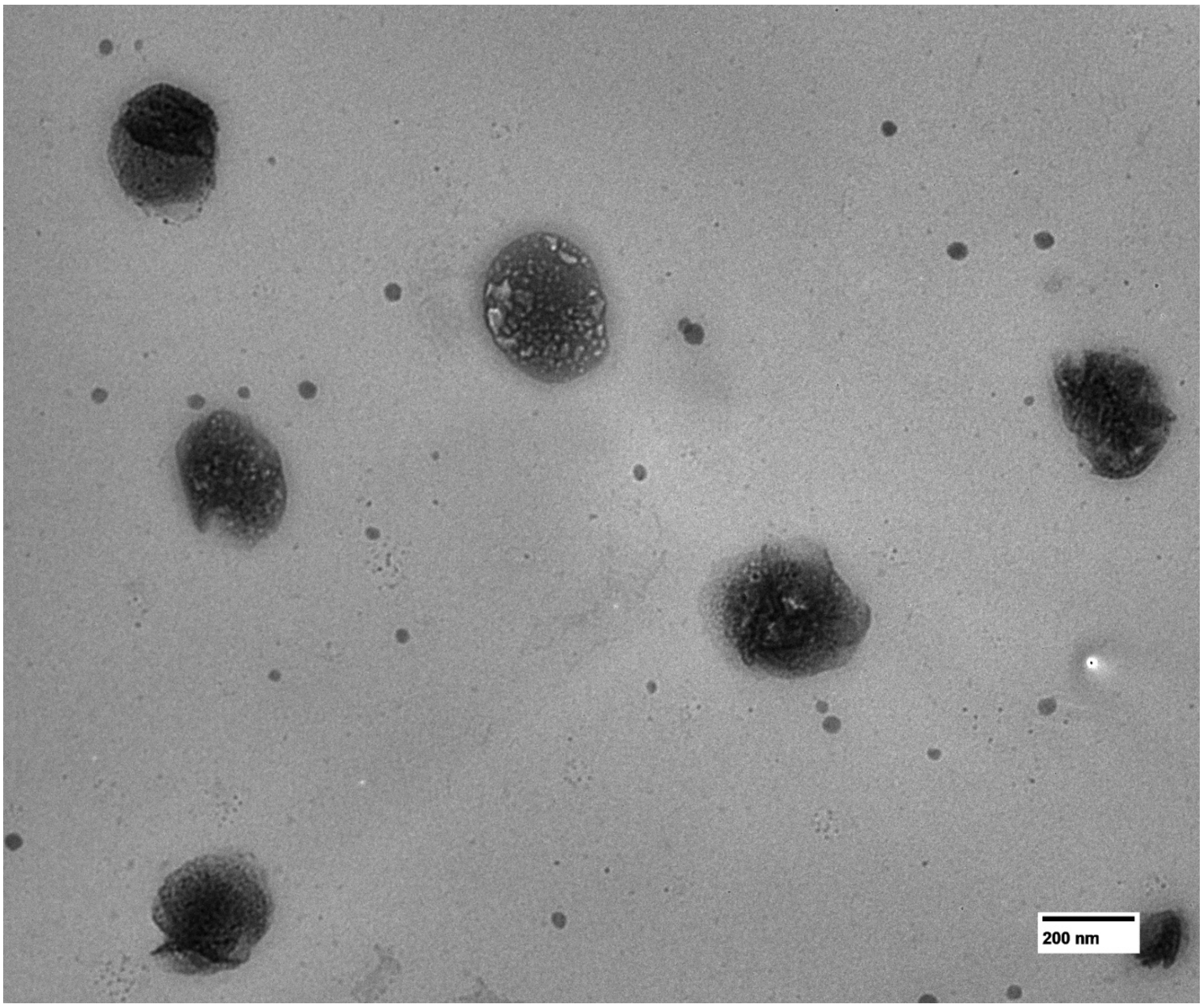

F2-B

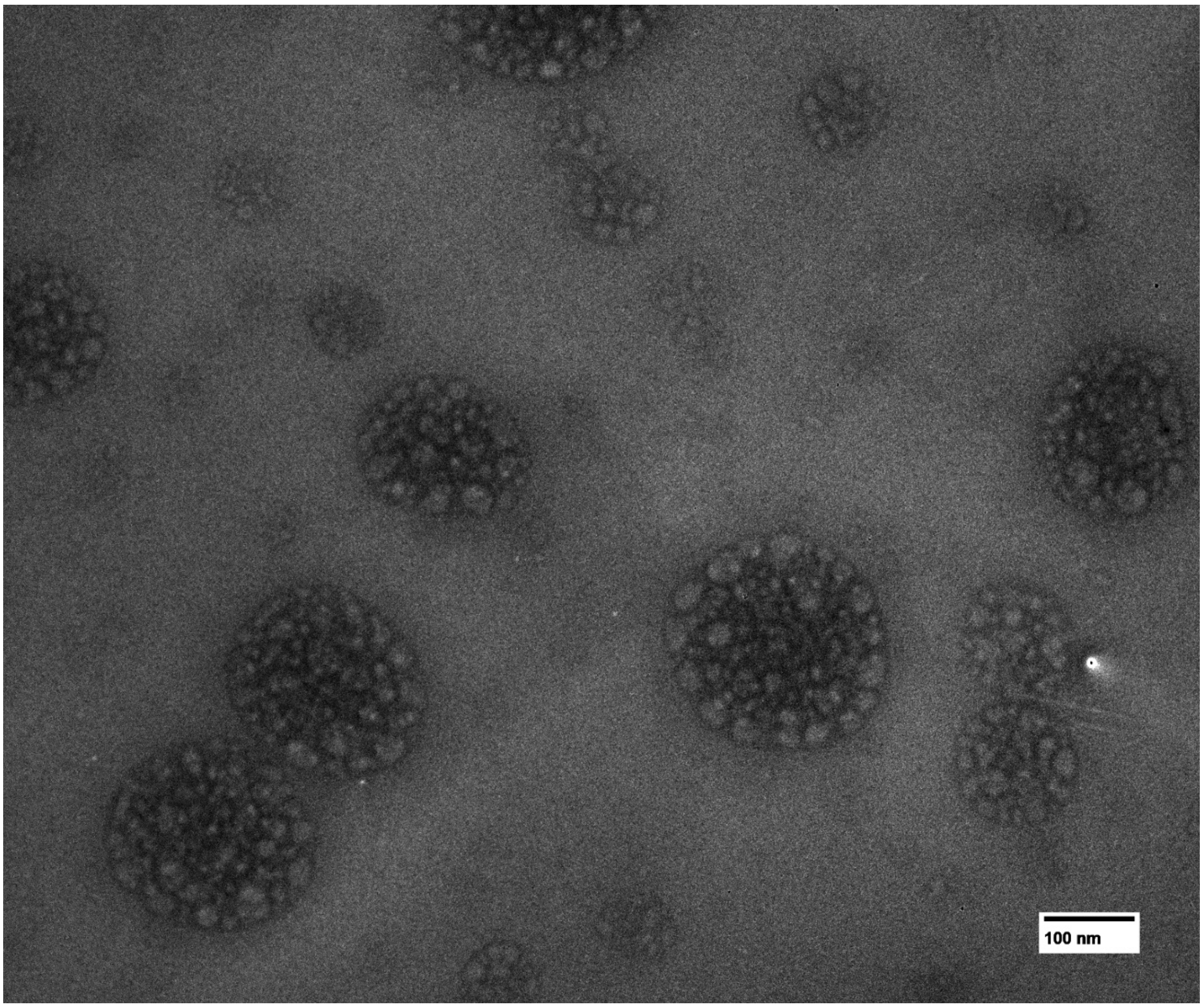

F1-Q4

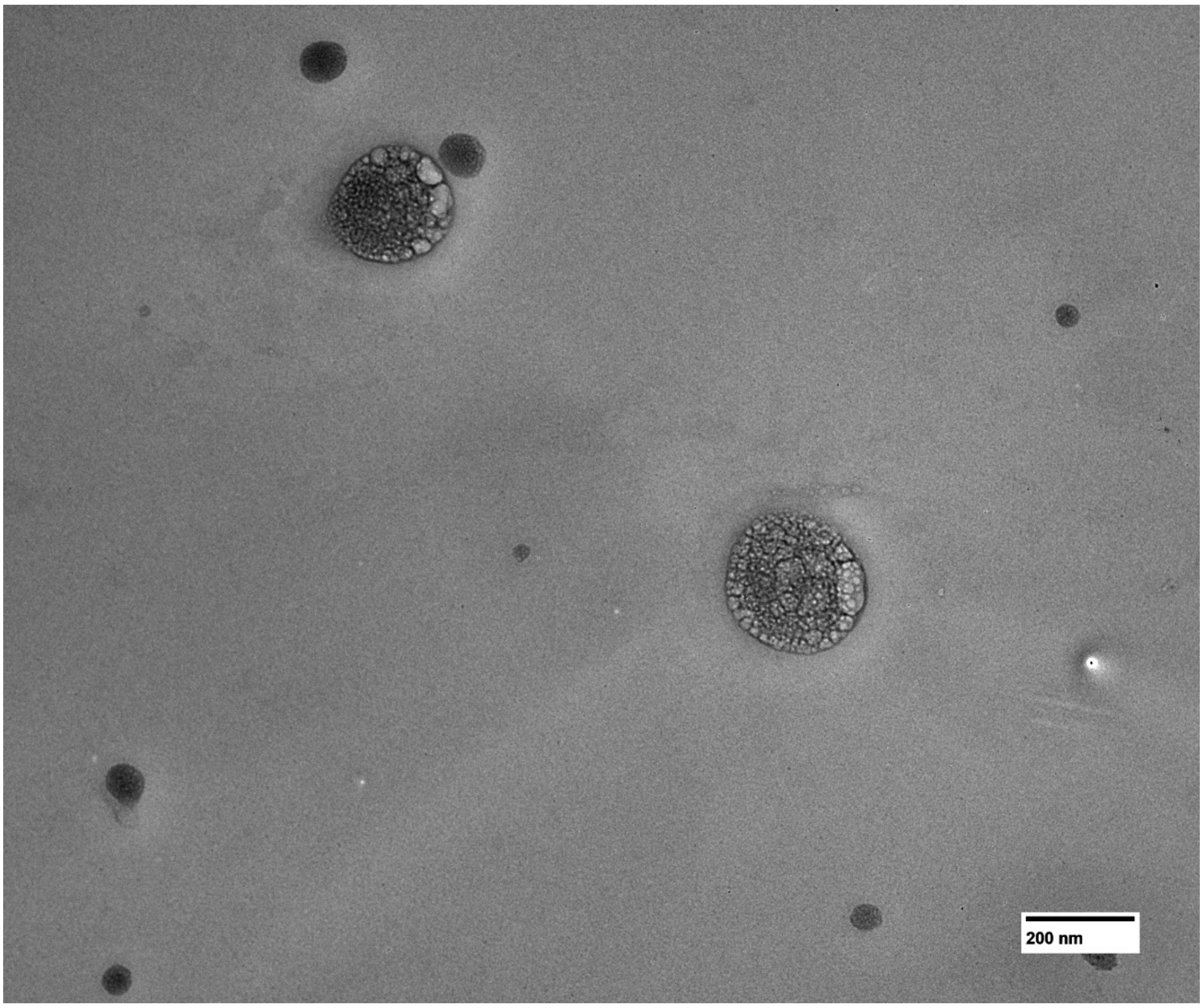

F2-Q4

Figure S3. Separate images obtained by Transmission Electron Microscopy (TEM) of the selected formulations F1-Q4 and F2-Q4, along with their corresponding quercetin-free systems (F1-B and F2-B). A scale bar of 100 nm was used for the F1 formulations (F1-B and F1-Q4), and a scale bar of 200 nm was used for the F2 formulations (F2-B and F2-Q4).

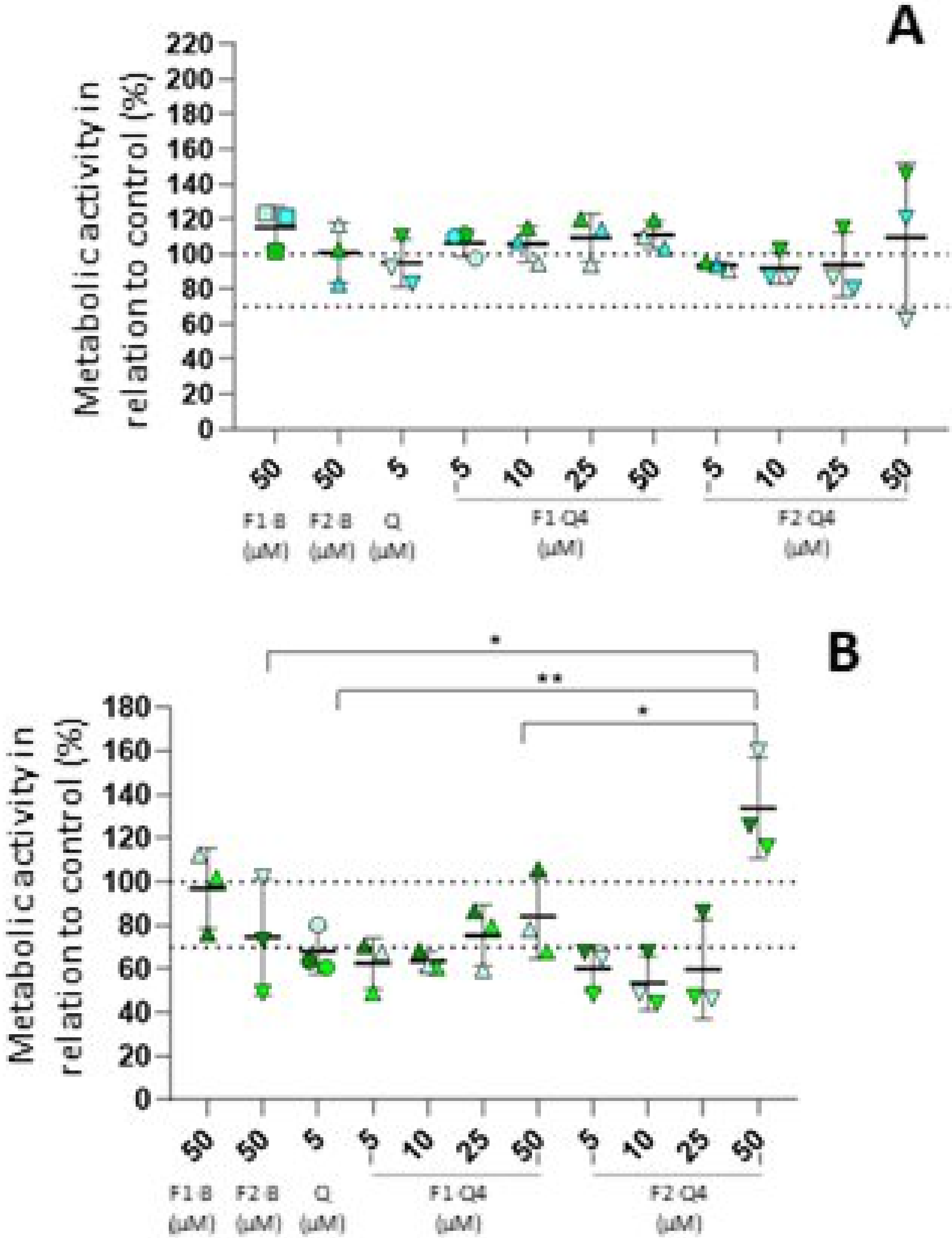
A
Metabolic activity in relation to control (%)
220
200
180
160
140
120
100
80
60
40
20
0
50
50
5
5
10
25
50
5
10
25
50
F1-B (μM)
F2-B (μM)
Q (μM)
F1-Q4 (μM)
F2-Q4 (μM)
B
*
**
*
Metabolic activity in relation to control (%)
180
160
140
120
100
80
60
40
20
0
50
50
5
5
10
25
50
5
10
25
50
F1-B (μM)
F2-B (μM)
Q (μM)
F1-Q4 (μM)
F2-Q4 (μM)

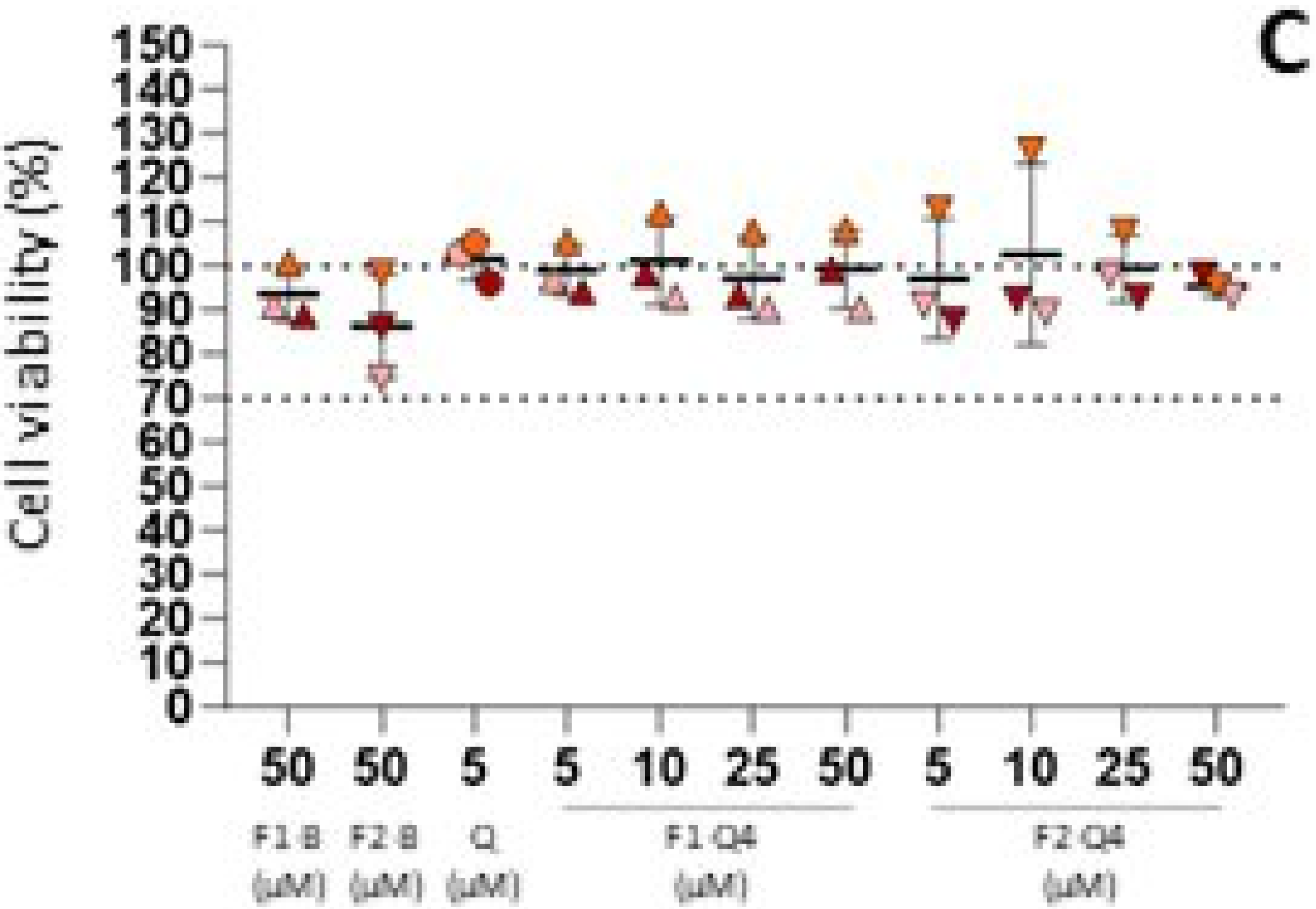
C
Cell viability (%)
150
140
130
120
110
100
90
80
70
60
50
40
30
20
10
0
50
50
5
5
10
25
50
5
10
25
50
F1-B (μM)
F2-B (μM)
Q (μM)
F1-Q4 (μM)
F2-Q4 (μM)

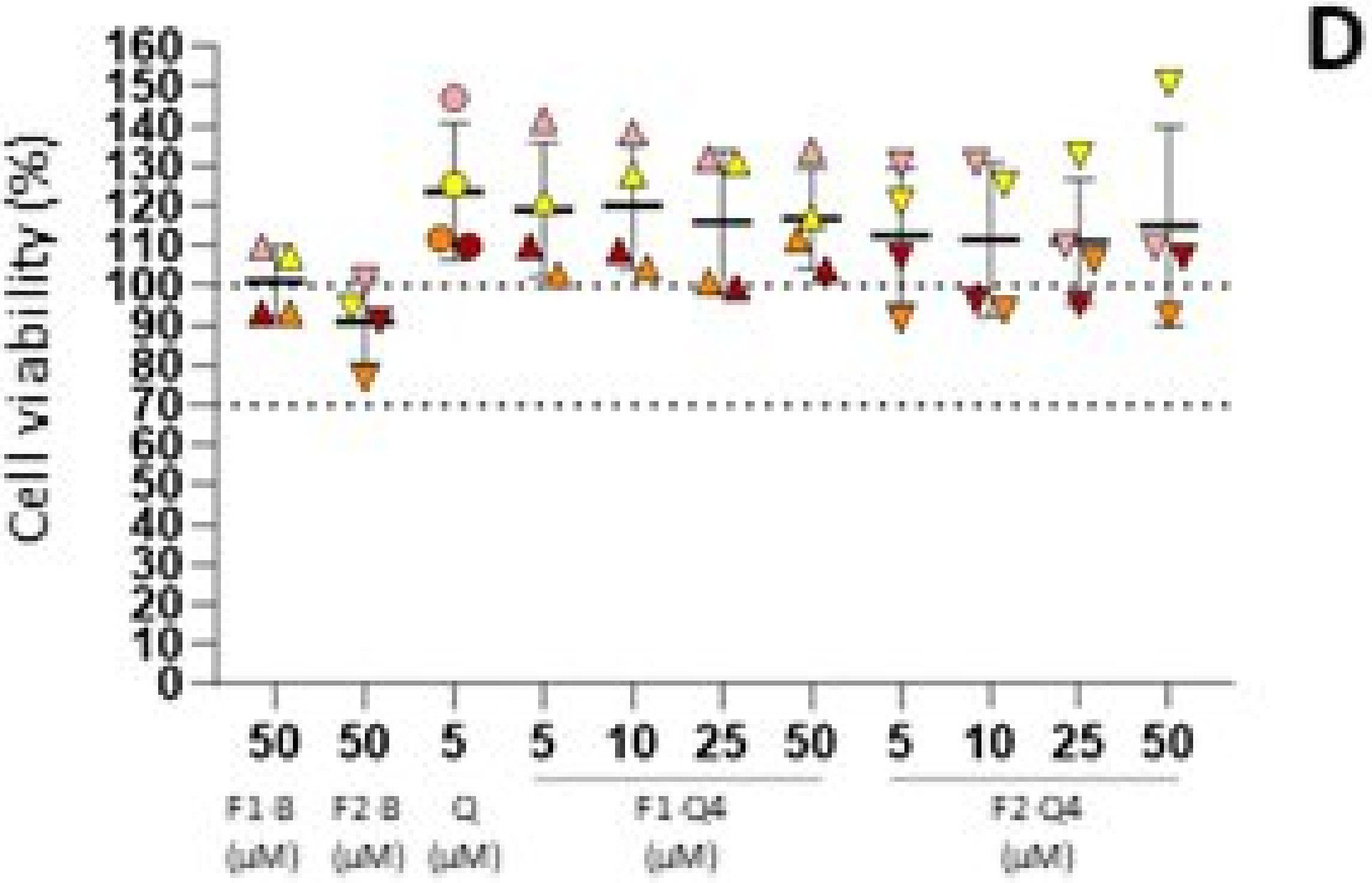
D
Cell viability (%)
160
150
140
130
120
110
100
90
80
70
60
50
40
30
20
10
0
50
50
5
5
10
25
50
5
10
25
50
F1-B (μM)
F2-B (μM)
Q (μM)
F1-Q4 (μM)
F2-Q4 (μM)

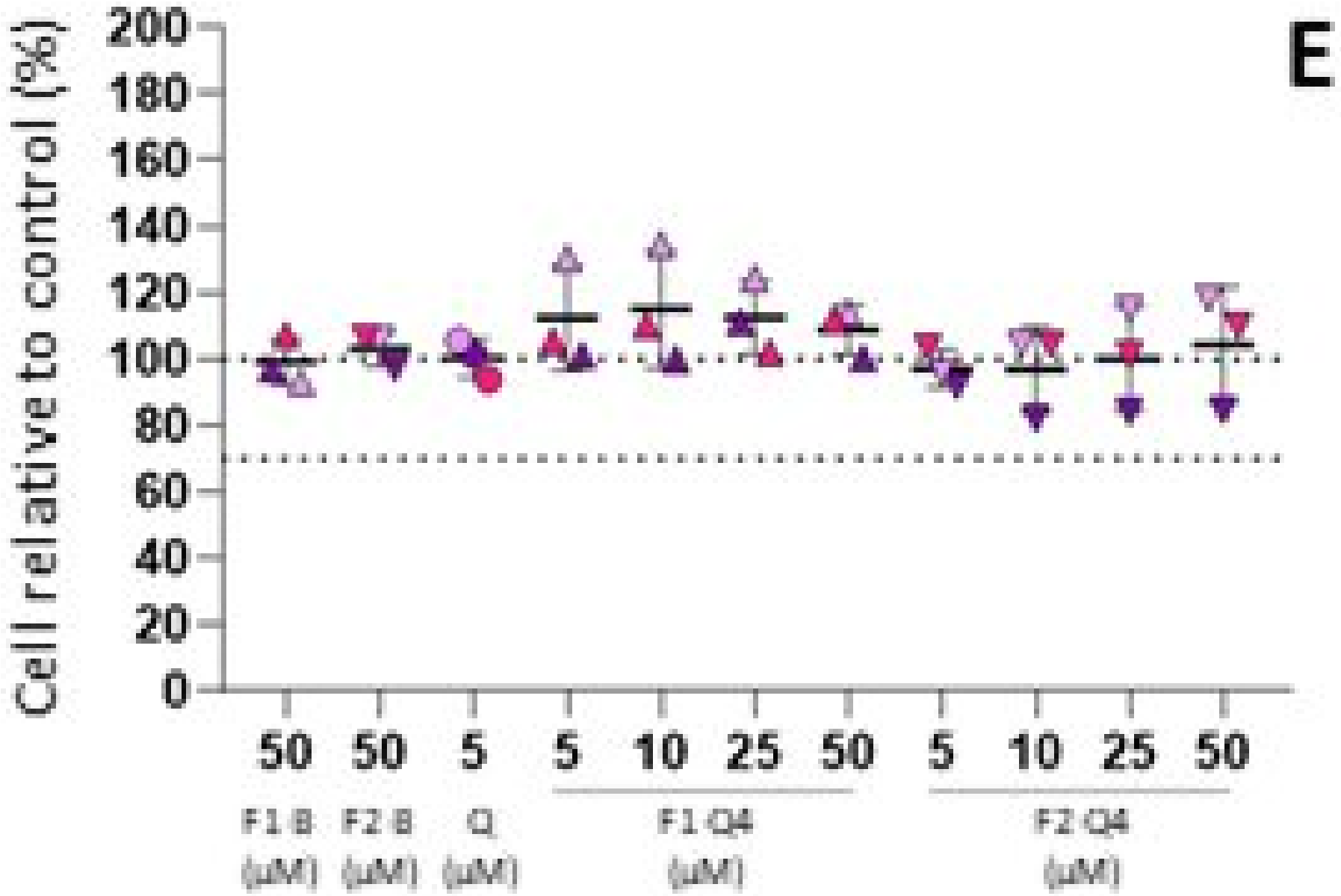

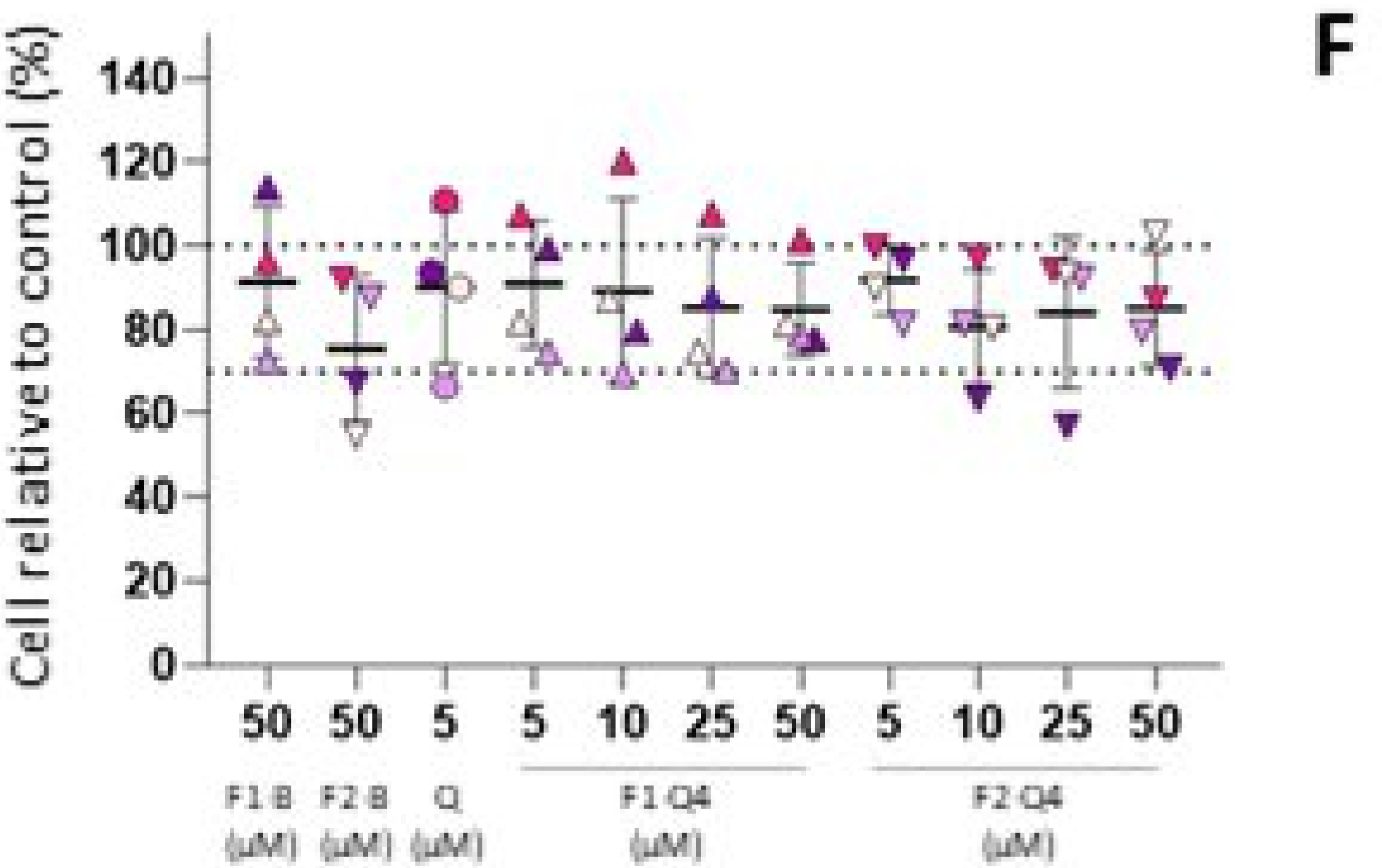

Figure S4. Separate images of the analysis of viability and cell proliferation of melanoma cells in the presence of free quercetin and quercetin-loaded formulations. The assays were carried out after 24 h of treatment. Metabolic activity was analyzed by MTT assay, cell viability by neutral red assay, and number of cells by violet crystal assay. * $p < 0.05$, ** $p < 0.01$ (ANOVA, Tukey – multiple comparisons between all groups).
